# Development of a culturally sensitive Arabic version of the Mini International Neuropsychiatric Interview (M.I.N.I.-AR) and validation of the depression module

**DOI:** 10.1186/s13033-021-00447-1

**Published:** 2021-03-18

**Authors:** Carine Karnouk, Kerem Böge, Nico Lindheimer, Dana Churbaji, Shaymaa Abdelmagid, Sara Mohamad, Eric Hahn, Malek Bajbouj

**Affiliations:** 1 Department of Psychiatry and Psychotherapy, Charité - Universitätsmedizin, Campus Benjamin Franklin, Berlin, Germany, corporate member of Freie Universität Berlin, Humboldt-Universität zu Berlin, and Berlin Institute of Health, Campus Benjamin Franklin, Hindenburgdamm 30, 12203 Berlin, Germany; 2 Balance Psychology Center, Cairo, Egypt

**Keywords:** Validation, MINI, Psychiatric diagnosis, Arabic, Cultural adaptation, Assessment, Arabic-speakers, Refugee

## Abstract

**Background:**

Arabic represents one of the most frequently spoken languages worldwide, especially among refugee populations. There is a pressing need for specialized diagnostic tools corresponding to the DSM-5 criteria in Modern Standard Arabic, which can be administered on Arabic speakers in the West and Arab region alike.

**Objectives:**

To develop and validate the culturally-adapted version of the most recent M.I.N.I. 7.0.2 into Modern Standard Arabic—a form of Arabic commonly used across all Arab countries.

**Methods:**

102 participants were recruited between April 2019 to March 2020 at the Charité - Universitätsmedizin in Berlin. Symptoms were assessed with Arabic versions of rater-based and self-rated measures, including Mini International Neuropsychiatric Interview (M.I.N.I.), Patient Health Questionnaire (PHQ-9), and Harvard Trauma Questionnaire (HTQ). Arabic-speaking psychiatrists saw participants for diagnostic assessment.

**Results:**

Cohen’s kappa (κ) values were moderate for major depression, and slight for post-traumatic stress disorder, as well as generalized anxiety disorder. Moreover, kappa values indicated moderate agreement between M.I.N.I.-AR and PHQ-9 for depression, as well as HTQ for post-traumatic stress disorder, respectively.

**Conclusion:**

The translated and culturally adapted version of the M.I.N.I. addresses an existing need for a reliable, efficient, and effective comprehensive diagnostic tool using the most recent DSM-5 criteria in Modern Standard Arabic (MSA). Based on the obtained results, only a validation of the depression module (Module A) of the M.I.N.I-AR was possible. Study outcomes also show evidence for the validation of Module H covering Post-Traumatic Stress Disorder. Potential valuable contributions can be extended to this translation and validation.

**Supplementary Information:**

The online version contains supplementary material available at 10.1186/s13033-021-00447-1.

## Introduction

There are as many as 274 million native Arabic speakers worldwide, making Arabic the 6th most spoken language [1]. Furthermore, 25 countries consider Arabic their official or co-official national language. Nowadays, Arabic is not only spoken in indigenous settings, but due to migration and refugee resettlements, Arabic-speakers now live dispersed globally [2, 3]. According to the German Federal Office for Migration and Refugees [4], Arabic is the most frequently spoken native language within Germany’s refugee population. In 2018, it was also reported that Arabic-speaking refugees had shown an increase in the prevalence of mental disorders [3] and are thus in need of accurate and efficient, culturally sensitive diagnostic assessments and treatment plans [2]. Furthermore, in Arabic-speaking countries, the burdens of mental health have been estimated to be much higher than in Western counterparts [5, 6]. Hence, Arabic-speakers face several disadvantages not only in their home countries but also in host countries and humanitarian aid settings [2, 7, 8]. Some of the challenges that they experience include lack of available effective and efficient culturally sensitive mental health services, cultural misunderstandings, stigma, and language barriers [2, 7, 8]. The aforementioned barriers have led to poor diagnoses and treatment choices.

Despite the high demand for specialized services for Arabic-speakers, a considerably large treatment gap exists [5, 9]. Furthermore, large-scale epidemiological studies and national data are scarce in the Arab region [10], making it challenging to estimate psychiatric prevalence rates and needs. In a study summarizing mental health services across the Arab region, it was found that there were less than 1 to 5 psychiatrists for every 100,000 inhabitants in 22 Arab countries [9]. Moreover, a comprehensive assessment using multiple search engines, found that in about 30 years (from 1966 to 1996), only a total of 1058 research papers were produced addressing the topic of mental health in the Arab world [10]. However, in the past decade, research contributions in the Arab world have reportedly increased by almost 160%, compared with only 57% for other regions. Although this number represents exponential growth (particularly in Egypt, Saudi Arabia and Lebanon), in total the number of research outputs is still comparably lower than the rest of the world [11]. The dearth of literature may explain the scarcity in available professional resources, assessment tools and specialized expertise in the region [5, 10], leading some Arab psychiatrists/psychologists to seek training in western contexts, thereby ignoring essential cultural pillars [8, 10]. As a result, over the years many unvalidated assessments and treatment methods have been adopted and used with Arab populations yielding to ‘culturally incongruent’ applications [2, 10]. However, in the last decade, significant progress has been made to address these issues in Arab and Western contexts resulting in increased research and hybrid mental health interventions taking elements from multiples cultures into consideration [7, 8, 10].

Clinical structured interviews were developed for diagnostic precision, speed and accuracy and carry several advantages. Because the questions are precise and elicit only a limited number of responses, these assessments can largely be administered by non-specialized interviewers, leading to high inter-rater reliability. Special algorithms are built to distinguish between clinically significant symptoms from regular stressors—a feature that can be used to ensure homogenous groups in clinical trials. Among the most commonly used clinical structured interviews internationally, are the Mini International Neuropsychiatric Interview (MINI) [12], Structured Clinical Interview (SCID), Present State Examination (PSE), Diagnostic Interview Schedule (DIS) and the Composite International Diagnostic Interview (CIDI) [13, 14]. From these interviews, only the DIS [15], the PSE-10 [16] and the CIDI [17], exist in Modern Standard Arabic, in addition to a Moroccan dialect validated version of the M.I.N.I. [18].

The M.I.N.I. was developed by Sheehan et al. [14] to explore some psychiatric disorders according to the DSM and ICD diagnostic criteria. Every few years, the M.I.N.I. is updated and validated versions in other languages also become available [14]. Initially, the M.I.N.I. was designed to meet the need for a short yet valid and reliable psychiatric interview for multicenter clinical trials and epidemiological studies. However, in recent years, the assessment tool is also being used in humanitarian aid and global health settings [7]. The M.I.N.I. is particularly attractive since it offers a fee waiver for researchers and clinicians who will use the assessment to assist refugees or victims of terrorism (for more information see https://harmresearch.org/index.php/mini-international-neuropsychiatric-interview-mini/). This stipulation may be more relevant now than ever, as refugee groups resettle worldwide.

The Moroccan version of the M.I.N.I. developed by Kadri et al. [18] corresponds to the DSM-IV criteria and was compared with expert diagnoses. This version uses the colloquial Moroccan dialect, which is quite different from Modern Standard Arabic and also includes French. The M.I.N.I. has been widely used in several studies in the Arab world, however, the majority of these studies developed their translations and focused on specific modules, such as post-partum depression [19], post-traumatic stress disorder [20], alcohol abuse and dependence [21] and schizophrenia [22, 23]. To the authors’ knowledge, there is no fully translated, culturally adapted and validated version of the M.I.N.I. available in Modern Standard Arabic (MSA) corresponding to the latest DSM-5 criteria.

This study aims to develop and validate a culturally-adapted version of the M.I.N.I. 7.0.2 in Modern Standard Arabic. Although various Arabic dialects exist, MSA—a standardized, formal version of the language, most commonly used in schools, the press and other official contexts—is widely understood by all native Arabic speakers.

## Methods

### Procedure and participants

The current cross-sectional validation study of the Arabic-version of the Mini International Neuropsychiatric Interview (M.I.N.I.-AR) was conducted to address the existing gap in available validated diagnostic assessment tools in Modern Standard Arabic language. Therefore, this adaption was developed in response to a need for the M.I.N.I.-AR to be used in a large German multi-center trial for Arabic speaking refugees in Germany (MEHIRA: Mental Health in Refugees and Asylum Seekers) [7]. The MEHIRA project depicts a multicenter randomized control trial investigating a stepped care and collaborative model (SCCM) for refugees and asylum seekers across eight university cities in Germany. All participants for the current trial were recruited through the similar recruitment channels of the MEHIRA network, which include regionally heterogeneous allocation paths, such as general practitioners, central clearing clinics, residential care settings, and social agencies in Berlin, Germany. Assessments took place either at the Charité - Universitätmedizin Berlin central clearing clinic, a specialized first-contact outpatient facility for refugees and asylum seekers in Berlin, or the Arabic-speaking outpatient clinic of the Department of Psychiatry and Psychotherapy, Campus Benjamin Franklin—both institutions led by the Charité - Universitätsmedizin in Berlin, Germany.

A sample of 102 participants was recruited between April 2019 to March 2020. Inclusion criteria were defined as age between 18 and 75 years and native Arabic speakers. Initially, two interviewers received in-depth structured training and supervision by two licensed psychologists to ensure consistency in the administration of the interview process. In the next step, participants received an information sheet about the study aims and were asked for their interest in participation. Throughout the whole study process, participants were encouraged to ask any questions that remain unclear. Participants who were interested and willing to participate gave their signed informed consent before any trial-related procedures were conducted. Study participation was voluntary, and no monetary compensation was provided. In the next step, participant symptomatology was assessed by using rater-based and self-rated measures, including the Mini International Neuropsychiatric Interview (M.I.N.I.), the Patient Health Questionnaire (PHQ-9), and the Harvard Trauma Questionnaire (HTQ). All assessments were conducted in Arabic. In parallel, participants were also seen by an Arabic-speaking licensed psychiatrist for a diagnostic assessment and consultation. Three separate psychiatrists were available in rotation at the outpatient clinic. Both interviewers and the psychiatrist were blind to the diagnosis of each other. The whole assessment procedure took between 60 and 90 min. The study was approved by the ethics committee of Charité - Universitätsmedizin Berlin, Germany, and is in line with the Declaration of Helsinki.

### The Mini International Neuropsychiatric Interview (M.I.N.I.)

For the current study, the M.I.N.I 7.0.2. validated English version was used and translated into Modern Standard Arabic (MSA, M.I.N.I.-AR; Additional file 1). The M.I.N.I. is a short (needing between 15 and 30 min for completion), structured diagnostic interview compatible with the DSM-5 and ICD-10 criteria. It was specifically designed for implementation in clinical practice and research in psychiatric-, as well as primary health care settings. The M.I.N.I. includes 130 questions with “yes” and “no” answer options examining 16 axis-I DSM-5 disorders as well as one personality disorder. Each of the 16 modules starts with a screening question to exclude the diagnoses and possibly skip the module accordingly if answered negatively or explore symptoms severity when responded positively. Several validation studies [12, 24] have demonstrated excellent interrater and test reliabilities of the M.I.N.I. Furthermore, moderate validity with both the extensive Composite International Diagnostic Interview (CIDI) [12, 14] and the Structured Clinical Interview for DSM-4 (SCID) [12, 14] have been exhibited. Through a previous trial by our research group with a pilot sample size (N = 20), initial validity has been shown for the translated M.I.N.I.-AR [25].

### Cultural adaptation and translation

**Fig. 1:**
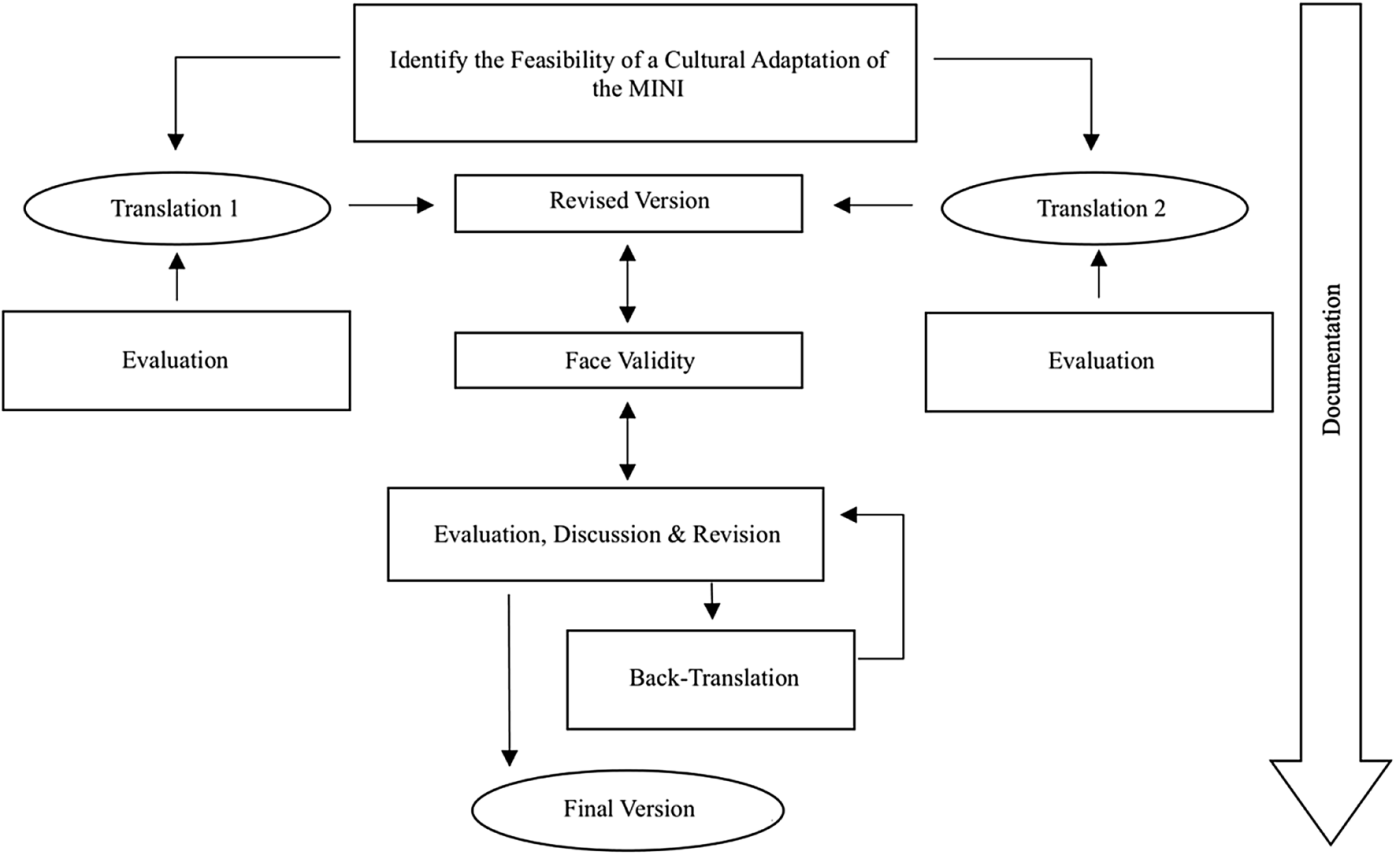
Illustrates the steps of the adaptation model of the MINI intro Modern Standard Arabic (MSA)

In a pilot trial published by our research group [25], a translation and cultural/linguistic adaptation of the M.I.N.I. 5.0.0 version was carried out. As a follow-up project, the validation of the most recent M.I.N.I. 7.0.2 version, with the same constellation of a multilingual, interdisciplinary team, including five psychologists and three psychiatrists, collaborated on a multilevel adaption process for the translation into the Arabic language (M.I.N.I.-AR). Members of the research group who were involved in the translation process are native Arabic speakers from different Arab nations. They have extensive experience in the mental health sector, as well as board knowledge and familiarity with the cultures of the original (English) and targeted language (Arabic). According to the World Health Organization guidelines [26], for the translation and adaptation process of instruments and aligned with Brislin [27], the steps of the adaption model are illustrated in Fig. 1.

In the first step, two translators (psychologists) evaluated the M.I.N.I. concerning its feasibility and practicability for a culturally-sensitive adaptation and translation. Secondly, both independently translated, assessed and revised all items. Afterward, potentially challenging and unclear items were discussed with a further psychologist (native English- and Arabic speaker) for accuracy and cultural relevance, and the translations were merged into one main revised version. To ensure face validity one trained interviewer according to the criteria by Lecrubier et al. [24], revised the version, and subsequently conducted it together with a licensed psychiatrist on Arabic speaking refugees as recommended by Bannigan and Watson [28].

Consequently, any unclear, problematic and challenging phrasing and linguistic expressions mentioned by the participants and the research staff were adjusted and adapted under special considerations of the Arab culture and cultural idioms within an iterative process of various feedback and revision loops (see example Table 1). Parallelly, a back-translation was performed into English by a blind, independent and bilingual psychologist to the original MINI-7.0 version according to international guidelines [26, 27]. In a final evaluation and revision, five psychologists and three psychiatrists discussed and adjusted outcomes until an agreement for a final version was reached. For a more detailed description of the cultural and linguistic adaptation and translation, see Churbaji et al. [25].

**Table 1:** Examples of the cultural adaptation of the MINI

Question MINI	Translation 1	Final translation	Type of equivalence	Explanation
Have you been consistently depressed or down most of the day, nearly every day, for the past two weeks?	هل شعرت بالحزن أو الاكتئاب بشكل مستمر، أغلب الوقت، بشكل يومي تقريباً، خلال الأسبوعين الماضيين؟	هل كنت تشعر بالحزن أو الاكتئاب بشكل مستمر، معظم اليوم، بشكل يومي تقريباً، طوال الأسبوعين الماضيين؟	Vocabulary equivalence	“down” ↔ “sad”. The term “down” can’t be used to describe emotions in the Arabic language
Did you have trouble sleeping nearly every night (difficulty falling asleep, waking up in the middle of the night, early morning wakening or sleeping excessively)?	هل واجهتك مشاكل في النوم، بشكل يومي تقريباً؟ (صعوبة في الخلود للنوم، أو الاستيقاظ المتكرر في منتصف الليل, أو الاستيقاظ بوقت باكر جداً, أو النوم بشكل مفرط)	هل واجهتك مشاكل في النوم, بشكل يومي تقريباً؟ (صعوبة في الخلود للنوم, أو الاستيقاظ المتكرر في منتصف الليل, أو الاستيقاظ في وقت باكر جداً, أو النوم بشكل مفرط)	Idiomatic equivalence	“falling asleep“ ↔ “go to sleep“. The term “falling asleep = غفوة” is used in the Arabic language for a nap
In the past 12 months, have you had 3 or more alcoholic drinks within a 3-h period on 3 or more occasions?	خلال الأشهر الـ ١٢ الماضية, هل شربت ثلاثة مشروبات كحولية أو أكثر, خلال ٣ ساعات، وحدث ذلك في ثلاث مناسبات أو أكثر؟	خلال الأشهر الـ ١٢ الماضية, هل شربت ثلاثة اواني كحولية أو أكثر, خلال ٣ ساعات، وحدث ذلك في ثلاث مناسبات أو أكثر؟	Experiential equivalence is not given	“alcoholic drinks” ↔ “alcoholic pots”. The quantity was not clearly understood. Even after adjustment, questions were asked about the size and the strength of the alcoholic drinks

### Questionnaires

The Patient Health Questionnaire-9 (PHQ-9) is a short and concise self-rating screening tool to assess the severity of depressive symptoms. It consists of nine items that cover all symptoms of a major depressive episode according to DSM-4 criteria [29]. Responses are given by participants on a four-point Likert scale (0 “not at all” to 3 “almost every day”), indicating their symptom severity for the past 2 weeks. Total scores can range from 0 to 27, with higher values indicating symptom severity. An overall score > 10 indicates the presence of depression. Cut-off values for symptoms severity are as following: minimal (2–9), mild (10–14), moderate (15–19), and severe (≥ 20). For the Arabic translation of the PHQ-9, a discriminant, factorial, and convergent validity and high reliability (0.86 ≤ α ≤ 0.88) have been shown [30].

The Harvard Trauma Questionnaire (HTQ) is a well-established self-reported screening instrument for trauma, torture, and post-traumatic stress disorder (PTSD) symptoms [31]. It covers three parts with 42 items, an open-ended question part, and 16 items, respectively. For the current study, only the third part was included to assess the severity of PTSD symptoms. Participants report their experiences with trauma symptoms such as “feeling detached or withdrawn from people”, “difficulty concentrating”, or “trouble sleeping” on a four-point Likert scale (1 “not at all” to 4 “extreme”) for each of the 16 items. A total score is calculated by dividing the sum of scores of items 1–16 by the number of items answered, with higher scores indicating an ascending level of PTSD symptom severity. The value can be between 1 and 4 with 2.5 as a cut-off value for a present PTSD, according to DSM-4 [31]. The scale has been validated in numerous cultures and languages, including Arabic, and has shown good psychometric properties [32, 33].

### Statistical analysis

All data was collected and stored pseudonymized in a spreadsheet using Package for the Social Science (SPSS) 25, MacOS-X. Statistical analyses were set at an exploratory significance level of *p* < 0.5. Descriptive measures of the sample are summarized as means and standard deviations (SD) for continuous measures and as frequencies and percentages for categorical variables. The criterion validity of the M.I.N.I.-AR was tested by comparing each M.I.N.I. diagnosis with the clinical diagnosis of a blind psychiatrist and its concordance with two screening tools namely the Patient Health Questionnaire 9 (PHQ-9) [29] for depressive symptoms (cut-off score set at 10), and the Harvard Trauma Questionnaire (HTQ) [34] (cut-off score set at 2.5) to detect the presence of post-traumatic stress symptoms. Numerous studies have highlighted that the assessment of criterion validity through the use of clinical diagnoses is a well-established method [13, 18]. Cohen’s kappa (κ) was used as a statistically adjusted measure of concordance between two measures [35]. It has been recommended that only for diagnoses with a frequency of 5% or more kappa values are calculated [13]. According to Landis and Koch [36] the following values were used to interpret significant results: < 0 no agreement, 0–0.20 slight, 0.21–0.40 fair, 0.41–0.60 moderate, 0.61–0.80 substantial, 0.81–1.0 perfect. Additionally, statistics for specificity, sensitivity, negative (NPV) and positive predictive values (PPV), and the number of false-positive and false-negative diagnoses were calculated.

## Results

### Clinical and sociodemographic characteristics

In total, the data of 102 Arabic-speaking refugee outpatients, 45 males, and 57 females, were analyzed. The mean age was 35.29 years (SD = 9.66; range 19–61). The vast majority of the sample named Syria as their country of origin (81%), followed by Iraq (7.8%) and Lebanon (4.5%). All sociodemographic characteristics assessed are illustrated in Table 2.

**Table 2:** Sociodemographic characteristics of the study sample

Sociodemographic data	*N* = 102
Gender	
Male	45 (44.1%)
Female	57 (55.9%)
Age in years M ± SD 35.29 ± 9.66*	*n* = 97
19–30	35 (34.3%)
31–40	35 (34.3%)
41–50	19 (18.6%)
51–61	8 (7.8%)
Country of origin	
Syria	81 (79.4%)
Iraq	8 (7.8%)
Lebanon	5 (4.9%)
Egypt	4 (3.9%)
Palestine	3 (2.9%)
Morocco	1 (1.0%)

Across all measures used, only major depressive episode or disorder (MDE) (M.I.N.I.: 83.3%, Expert: 61.5%, PHQ-9: 74.5%), posttraumatic stress disorder (PTSD) (M.I.N.I.: 36.3%, Expert: 14.6%, HTQ: 52.0%), and generalized anxiety disorder (M.I.N.I.: 36.6%, Expert: 5.2%) had a occurance of < 5% in the M.I.N.I. screening as well as expert diagnoses. Detailed prevalence estimates for each disorder of the present sample by at least one of the measures assessed is depicted in Table 3.

**Table 3:** Prevalence estimates, depending on the measure used

Prevalence estimates	M.I.N.I	Expert	PHQ/HTQ
	*N* = 102	*N* = 96	*N* = 102
A. Major depressive episode/disorder	78 (76.5%)*	59 (61.5%)	76 (74.5%)
C. Manic episode/bipolar disorder	2 (2.0%)	1 (1.0%)	
D. Panic disorder	34 (33.3%)	3 (3.1%)	
E. Agoraphobia	12 (11.8%)	0 (0.0%)	
F. Social anxiety	14 (13.7%)	4 (4.2%)	
G. Obsessive–compulsive disorder	16 (15.7%)	2 (2.1%)	
H. Post-traumatic stress disorder	37 (36.3%)	13 (13.5%)	53 (52.0%)
I. Alcohol use disorder	3 (2.9%)	3 (3.1%)	
J. Substance use disorder	3 (2.9%)	3 (3.1%)	
K. Psychotic disorder	0 (0.0%)	1 (1.0%)	
L. Anorexia nervosa	1 (1.0%)	0 (0.0%)	
M. Bulimia nervosa	1 (1.0%)	0 (0.0%)	
MB. Binge-eating disorder	0 (0.0%)	0 (0.0%)	
N. Generalized anxiety disorder	11 (10.8%)	9 (9.4%)	
P. Antisocial PS	7 (6.9%)	0 (0.0%)	
Adjustment disorder		12 (12.5%)	
Somatoform disorders		3 (3.1%)	
Dissociative disorders		3 (3.1%)	
Sexual dysfunctions		1 (1.0%)	
Emotionally unstable PS		1 (1.0%)	
Hyperkinetic disorders		1 (1.0%)	
Nonorganic sleep disorders		1 (1.0%)	
Histrionic PS		1 (1.0%)	
Pathological gambling		1 (1.0%)	

*Excluding 7 past episodes. Multiple diagnoses per individual are possible

*M.I.N.I.* Mini International Neuropsychiatric Interview, *Expert* clinical expert diagnoses, *PHQ* Patient Health Questionnaire 9, *HTQ* Harvard Trauma Questionnaire, *PS* personality disorder

### M.I.N.I.-AR concordance with clinical diagnoses

A total of 96 individuals received a diagnosis from both the M.I.N.I.-AR and a clinical expert. Since only major depressive episode, post-traumatic stress disorder, and generalized anxiety disorders had a prevalence of < 5% in the M.I.N.I. screening and expert diagnoses, all other diagnoses were excluded from the validity assessment. Generally, kappa values were moderate for major depression episodes/disorders (0.54) and slight for post-traumatic stress disorders (0.2), and generalized anxiety disorder (0.12). All results for the three disorders are depicted in Table 4.

**Table 4:** Concordance of the M.I.N.I.-AR with clinical diagnoses

Disorder		Expert diagnosis			Kappa (95% CI)	Sensitivity (%) (95% CI)	Specificity (%) (95% CI)	PPV (%) (95% CI)	NPV (%) (95% CI)
	M.I.N.I.		−	+					
		−	TN	FN					
		+	FP	TP					
Major depressive episode/disorder			19	1	0.54 (0.38 to 0.71)	98.31 (90.91 to 99.96)	51.35 (34.40 to 68.08)	74.32 (67.79 to 81.80)	95.00 (72.63 to 99.27)
			18	58					
Post-traumatic stress disorder			55	4	0.20 (0.04 to 0.37)	69.23 (38.57 to 90.91)	66.27 (55.05 to 76.28)	24.32 (16.71 to 33.99)	93.22 (85.71 to 96.93)
			28	9					
Generalized anxiety disorder			79	7	0.12 (− 0.13 to 0.39)	22.22 (2.81 to 60.01)	90.86 (82.68 to 95.95)	20.00 (5.87 to 50.07)	91.86 (88.78 to 94.15)
			8	2					

*N* = 96

*M.I.N.I.* Mini International Neuropsychiatric Interview, *Expert* clinical expert diagnoses, *CI* confidence interval, *TP* true positive, *FP* false positive, *FN* false negative, *TN* true negative, *PPV* positive predictive value, *NPV* negative predictive value

### M.I.N.I.-AR concordance with the PHQ-9 and HTQ

102 participants were administered the M.I.N.I.-AR and filled self-rating measures PHQ-9 and HTQ. Kappa values indicated moderate agreement between M.I.N.I.-AR and PHQ-9 (0.58) as well as HTQ (0.53), respectively. Detailed results are summarized in Table 5.

**Table 5:** Concordance of the M.I.N.I.-AR with the PHQ-9 and the HTQ

Disorder		PHQ-9/HTQ			Kappa (95% CI)	Sensitivity (%) (95% CI)	Specificity (%) (95% CI)	PPV (%) (95% CI)	NPV (%) (95% CI)
	M.I.N.I.		−	+					
		−	TN	FN					
		+	FP	TP					
Major depressive episode/disorder			17	7	0.58 (0.39 to 0.76)	90.79 (81.94 to 96.22)	65.38 (44.33 to 82.79)	88.46 (81.81 to 92.89)	70.83 (53.20 to 83.84)
			9	69					
Post-traumatic stress disorder			45	20	0.53 (0.38 to 0.69)	62.26 (47.89 to 75.21)	91.84 (80.40 to 97.73)	89.19 (75.91 to 95.57)	69.23 (61.19 to 76.25)
			4	33					

*N* = 102

*M.I.N.I.* Mini International Neuropsychiatric Interview, *Expert* clinical expert diagnoses, *CI* confidence interval, *TP* true positive, *FP* false positive, *FN* false negative, *TN* true negative, *PPV* positive predictive value, *NPV* negative predictive value

## Discussion

The overall aim of the current study was to validate a translated and culturally adapted version of the most recent M.I.N.I. 7.0.2 into Modern Standard Arabic—a form of Arabic commonly used across all Arab countries in official settings and the press. Although the obtained results did not show evidence for the validation of all the modules within the instrument, it was possible to validate the depression module (Module A). Results showed moderate agreements between Module A of the M.I.N.I.-AR 7.0.2 compared with expert diagnosis and the PHQ. Also noteworthy to reveal were the moderate to slight agreements between M.I.N.I.-AR, HTQ and expert diagnosis concerning post-traumatic stress disorder. The M.I.N.I.-AR 7.0.2 was translated and culturally-adapted to address a diagnostic gap in a large German research trial for the psychological treatment of refugees of Arab descent [7]. Together with our pilot trial [25], this Arabic version of the M.I.N.I. can contribute to the existing literature and be used in mental health care settings serving Arabic-speaking populations as a whole. The adapted instrument is relevant to both Arab and Western contexts alike. Nonetheless, further studies and extensions may use this carefully adapted translation and compare it with a gold standard structured interview that may offer more robust results.

Our study outcomes are in line with a similar validation study using the same method [13] and our initial pilot study [25]. The original authors of the M.I.N.I. also reported a rather poor agreement between expert diagnoses and M.I.N.I. diagnoses in their validation studies [14]. This is not surprising, since the M.I.N.I. was originally designed to be oversensitive. As reported by Sheehan et al. [14], over-diagnosing cases may be less harmful than missing a case. One explanation could be that experts may not be able to review all the information available and thus overlook diagnoses [37]. In contrast, the M.I.N.I. is a particularly comprehensive tool offering fast, efficient and accurate diagnoses. This is a particular asset of the M.I.N.I. since it can be administered by non-specialists leading to resource-saving and cost-effectiveness—both highly relevant benefits, especially in the case of Europe’s refugee resettlements and the scarcity of resources and qualified personnel in the Arab world.

Recent studies have highlighted that patients from a Muslim cultural background may experience feelings of discomfort when alone with a therapist from the opposite gender [38], sometimes resulting in an inaccurate representation of symptoms during psychiatric evaluation. In the present study, gender may have played a role in participant responses. Matching gender between psychiatrists and patients was not always possible in the expert interviews, whereas there was more flexibility in the administration of the M.I.N.I.. Since the M.I.N.I. was designed to be administered by non-specialists, it provides an efficient solution to the shortage of available specialized professional care, who sometimes lack the language skills and cultural competence training needed in diagnosing Arabic-speaking populations. Taken together, these reports may explain the slight to moderate Kappa values when comparing the M.I.N.I.-AR and expert diagnoses. Nonetheless, the specific effect of matching gender was not within the scope of this paper, however, it may be interesting to assess this in future validation studies.

The present study has several strengths, especially when considering the limited availability of diagnostic tools and research targeting this specific population. A key strength of the present study is the careful translation and cultural adaptation process of the M.I.N.I. 7.0.2 into Modern Standard Arabic. As mentioned above, Modern Standard Arabic is particularly useful since it is a form of Arabic that is pluricentric—a major practical advantage for Arabic speakers and clinicians worldwide. Language and cultural barriers are one of the factors contributing to the underrepresentation of minorities in clinical settings and research samples [39]. This instrument can thus be used to increase the representation of minorities from the Arab region in clinical practice and research. The translation and adaptation process of the M.I.N.I. was based on experiences from our pilot study [25] and included the same multilingual and interdisciplinary team of psychologists and psychiatrists, each with their unique set of expertise. The team included Arabic-speakers from different Arab countries. The translation and adaptation followed the WHO guidelines [26] and were in line with the adaptation model developed by Brislin [27]. Furthermore, the use of culturally appropriate stories or metaphors to emphasize key concepts helps keep patients engaged and can be used in the diagnosis and different stages of the therapeutic encounter [40]. Sometimes, patients are unable to name an illness to describe their condition—in some languages, words used to name emotions may not correspond to those used in psychiatric settings in the West. For example, using words such as feeling “down” or “high” to describe an emotion does not exist in Arabic. Careful consideration was given to the meaning of the translation to ensure exact understanding. Therefore, by including culturally appropriate words to describe emotions, this instrument can improve the quality of diagnosis and therapeutic encounters when applied with Arabic-speakers and refugee communities in the West [38] and in the Arab region. The M.I.N.I-AR also contains a comprehensive table of drugs that are commonly used in the Arab region including their local street names.

The findings of this study have to be seen in the light of several limitations. To conduct a robust validation, a gold standard structured interview coupled with a larger sample size and a more clinically diverse sample is needed. This was not possible due a scarcity of resources (clinic capacity), and the vulnerability of our sample (difficultry concentrating and fatigue), therefore valuable future contributions can be extended to this validation. Furthermore, study results show a high count of adjustment disorders in expert diagnoses, but no possibility to diagnose this with the M.I.N.I. Indeed, this is a weakness of the M.I.N.I. In many settings, the diagnosis of adjustment disorder seems less stigmatizing and more easily acceptable [41] and may thus be especially appropriate for individuals from the Arab world, where mental health stigma plays an important role [42]. Nonetheless, similar symptoms are present in depression and PTSD, so they are accounted for. A further limitation is a comparison of HTQ and PHQ (DSM-IV) with the M.I.N.I. trauma section and depression sections (DSM-5). Even though there was a considerable amount of changes from the DSM-IV to 5 in the definition of trauma [43], and rather small changes in the depression section [44], the questions in the M.I.N.I. versions remained mostly the same and should thus be appropriate for the comparison with questionnaires using the DSM-IV as a basis. This may also be a consideration for future research. Moreover, study results also revealed a low prevalence of general disorders. In our sample, the most prevalent diagnoses were depression, PTSD, and anxiety; therefore, reliable kappa values could not be calculated for many disorders. Yet, the proportion of disorders in the current study mirrors the prevalence of the most reported disorders in the refugee community [45], reflecting ecological validity. The M.I.N.I-AR was validated on a sample made up of mostly young Syrians, and Arab patients living in the West may differ from those living in their country of origin [38]. Therefore, for generalizability, future directions for this research may target Arab populations with a more diverse representation of psychiatric disorders.

In conclusion, our translated and culturally-adapted M.I.N.I-AR addresses an existing gap and need for a reliable, efficient, and effective diagnostic tool that can be used in both clinical and research settings involving Arabic speakers worldwide. Based on the obtained results, we were only able to validate module A of the M.I.N.I-AR covering depression and show some evidence in support of the validation of Module H covering Post-Traumatic Stress Disorder. Since no other diagnostic tool corresponding to the DSM-5 criteria is currently available in Modern Standard Arabic, the present translation and cultural adaptation particularly useful for Western host countries and humanitarian aid settings that are unable to find an alternative. Although the instrument was validated on a sample of Syrian refugees, it was developed in Modern Standard Arabic, leading to its potential usefulness in the entire Arab region.

## Supplementary Information

**Additional file 1.** M.I.N.I.-AR.

## Data Availability

The dataset used and analysed in the current study is available upon request from the corresponding author.
