# Supplementary material for: Development of a culturally sensitive Arabic version of the Mini International Neuropsychiatric Interview (M.I.N.I.-AR) and validation of the depression module
Source: Int J Ment Health Syst. 2021 Mar 18;15:24. doi: 10.1186/s13033-021-00447-1 (PMC7977598; doi:10.1186/s13033-021-00447-1)
Supplement: Supplementary file 1 — Additional file 1. M.I.N.I.-AR. [file 13033_2021_447_MOESM1_ESM.pdf]

M.I.N.I

MINI International Neuropsychiatric Interview

Arabic Version 7.0.2

For

DSM-5

© Copyright 1992-2016 Sheehan DV
All rights reserved. No part of this document may be reproduced or transmitted in any form, or by any means, electronic or mechanical, including photocopying, or by any information storage or retrieval system, without permission in writing from Dr.
Sheehan. Individual researchers, clinicians and students working in nonprofit or publicly owned settings (including universities, nonprofit hospitals, and government institutions) may make paper copies of a M.I.N.I. instrument for their personal clinical and
research use, but not for institutional use, or for any financial profit or gain. Any use involving financial gain requires a license agreement from the copyright holder and payment of a per use license fee.

DISCLAIMER

Our aim is to assist in the assessment and tracking of patients with greater efficiency and accuracy. Before action is taken on any data collected and processed by this program, it should be reviewed and interpreted by a licensed clinician.

This program is not designed or intended to be used in the place of a full medical and psychiatric evaluation by a qualified licensed physician – psychiatrist. It is intended only as a tool to facilitate accurate data collection and processing of symptoms elicited by trained personnel. It is not a diagnostic test.

© 2019 Charité Universitätsmedizin Berlin - Campus Benjamin Franklin. All rights reserved. Translated by Dana Churbaji, Shaymaa Hauary, Sara Mohamed & Carine Karnouk. Supervised by Dr. Malek Bajbouj.

رقم المريض:	اسم المريض:
موعد بداية المقابلة:	تاريخ الميلاد:
موعد نهاية المقابلة:	اسم القائم بإجراء المقابلة:
الوقت الكلي المقابلة:	تاريخ المقابلة:

التشخيص الأولي	ICD-10-CM	تم استيفاء المعايير	الإطار الزمني للاضطراب	Modules - الوحدات	
		☐	حالية (أسبوعان)	نوبة اكتئاب كبرى<br>MAJOR DEPRESSIVE EPISODE	A
		☐	ماضية		
		☐	متكررة الحدوث		
☐	F32.x	☐	حالي (أسبوعان)	اضطراب الاكتئاب الرئيسي<br>MAJOR DEPRESSIVE DISORDER	
☐	F32.x	☐	ماضي		
☐	F33.x	☐	متكرر الحدوث		
☐		☐	حالي (الشهر الماضي)	الانتحار<br>SUICIDALITY	B
☐	عالي ☐<br>متوسط ☐<br>منخفض ☐	☐	محاولات مزمنة مدى الحياة		
☐	السنة الماضية	☐	حالي	اضطراب السلوك الانتحاري<br>SUICIDE BEHAVIOR DISORDER	
☐	١-٢ سنة الماضية	☐	في مرحلة تعافي مبكر		
		☐	حالية	نوبة هوس خفيف<br>MANIC EPISODE	C
		☐	ماضية		
		☐	حالية	نوبة هوس<br>HYPOMANIC EPISODE	
	لم يتم استكشافها ☐	☐	ماضية		
☐	F31.0 - F31.76	☐	حالي	اضطراب ثنائي القطب I<br>BIPOLAR I DISORDER	
☐	F31.0 - F31.76	☐	ماضي		
☐	F31.2/31.5/F31.64	☐	حالي	اضطراب ثنائي القطب I مع ملامح ذهانية<br>BIPOLAR I DISORDER WITH PSYCHOTIC FEATURES	
☐	F31.2/31.5/F31.64	☐	ماضي		
☐	F31.81	☐	حالي	اضطراب ثنائي القطب II<br>BIPOLAR II DISORDER	
☐	F31.81	☐	ماضي		
☐	F31.89	☐	حالي	اضطرابات أخرى ذات صلة باضطراب ثنائي القطب<br>OTHER SPECIFIED BIPOLAR AND RELATED DISORDER	
☐	F31.89	☐	ماضي		
☐	F41.0	☐	حالي (الشهر الماضي)	اضطراب الهلع<br>PANIC DISORDER	D
☐	F40.0	☐	مزمن مدى الحياة		
☐	F40.00	☐	حالي	رهاب الأماكن المفتوحة/العامة<br>AGORAPHOBIA	E
☐	F40.10	☐	حالي (الشهر الماضي)	اضطراب القلق الاجتماعي (الرهاب الاجتماعي)<br>SOCIAL ANXIETY DISORDER (Social Phobia)	F
☐	F42.2	☐	حالي (الشهر الماضي)	اضطراب الوسواس القهري<br>OBSESSIVE-COMPULSIVE DISORDER	G
☐	F43.10	☐	حالي (الشهر الماضي)	اضطراب كرب ما بعد الصدمة<br>POSTTRAUMATIC STRESS DISORDER	H
☐	F10.10 - F10.21	☐	١٢ شهر الماضية	اضطراب تعاطي الكحول<br>ALCOHOL USE DISORDER	I
☐	F11.10 - F19.21	☐	١٢ شهر الماضية	اضطراب تعاطي المواد المخدرة (الغير كحولية)<br>SUBSTANCE USE DISORDER (Non-alcohol)	J
☐	F20.81-F29	☐	حالي	أي اضطرابات ذهانية<br>ANY PSYCHOTIC DISORDER	K
☐	F20.81-F29	☐	مزمن مدى الحياة		

© 2019 Charité Universitätsmedizin Berlin - Campus Benjamin Franklin. All rights reserved. Translated by Dana Churbaji, Shaymaa Hauary, Sara Mohamed & Carine Karnouk. Supervised by Dr. Malek Bajbouj.

	نوبة اكتئابية مع ملامح ذهانية<br>MAJOR DEPRESSIVE DISORDER WITH PSYCHOTIC FEATURES	حالية	☐	F32.3/F33.3	☐
		مزمن مدى الحياة	☐	F32.3/F33.3	☐
	اضطراب ثنائي القطب مع ملامح ذهانية<br>BIPOLAR I DISORDER WITH PSYCHOTIC FEATURES	حالي	☐	F31.2/F31.5/F31.64	☐
		مزمن مدى الحياة	☐	F31.2/F31.5/F31.64	☐
L	فقدان الشهية العصبي<br>ANOREXIA NERVOSA	حالي (آخر ٣ أشهر)	☐	F50.01/F50.02	☐
M	الشره العصبي<br>BULIMIA NERVOSA	حالي (آخر ٣ أشهر)	☐	F50.2	☐
MB	اضطراب نوبات الأكل الشره<br>BINGE-EATING DISORDER	حالي (آخر ٣ أشهر)	☐	F50.81	☐
N	اضطراب القلق العام<br>GENERALIZED ANXIETY DISORDER	حالي (آخر ٣ أشهر)	☐	F41.1	☐
O	استبعاد المسببات الطبية، العضوية, المرتبطة بالعقاقير أو المخدرات<br>MEDICAL, ORGANIC, DRUG CAUSE RULED OUT		☐<br>نعم	☐<br>لا	☐<br>غير مؤكد
P	اضطراب الشخصية المعادية للمجتمع<br>ANTISOCIAL PERSONALITY DISORDER	مزمن مدى الحياة	☐	F60.2	☐

حدد التشخيص الأولي عن طريق اختيار المربع المناسب للتشخيص

(ما هي المشكلة التي تزعجك أكثر أو تفوق المشاكل الأخرى أو ظهرت بشكل أبكر في تاريخك المرضي؟)

© 2019 Charité Universitätsmedizin Berlin - Campus Benjamin Franklin. All rights reserved. Translated by Dana Churbaji, Shaymaa Hauary, Sara Mohamed & Carine Karnouk. Supervised by Dr. Malek Bajbouj.

التعليمات والإرشادات العامة

تم تصميم M.I.N.I. كمقابلة مُنظمة مُختصرة للاضطرابات النفسية الرئيسية في النسخة الخامسة من الدليل التشخيصي والإحصائي للاضطرابات النفسية والعقلية DSM-5 وفي دليل التصنيف الدولي للأمراض ICD-10. وقد أجريت عدة دراسات لمضاهاة مدي الثبات والصدق بين الـ M.I.N.I. وبينP-SCID لـ R-III-DSM وCIDI (وهي مقابلة منظمة قد تم تصميمها من قبل منظمة الصحة العالمية). وقد أظهرت نتائج هذه الدراسات أن الـ M.I.N.I. له خصائص مماثلة لمعايير الثبات والصدق، ولكن يُمكن أن يتم تطبيقه في فترة زمنية أقصر بكثير (متوسط 18.7 ± 11.6 دقيقة، متوسط 15 دقيقة) من الأدوات الأخرى المشار إليها أعلاه. ويُمكن للأطباء النفسيين والاختصاصيين في المجال النفسي الإكلينيكي استخدامه، بعد دورة تدريبية قصيرة. ولكن يحتاج غير المُتخصصين في مجال الصحة النفسية إلى التدريب المُكثف للقيام بتطبيقه.

المقابلة: من أجل الحفاظ على المقابلة قصيرة قدر الإمكان، قُم بإبلاغ المريض أنك ستجري مقابلة إكلينيكية أكثر تنظيما من المعتاد، مع طرح أسئلة دقيقة للغاية حول المشكلات النفسية التي تتطلب إجابة نعم أو لا.

الشكل العام: ينقسم M.I.N.I إلى وحدات محددة بأحرف، كل منها مطابق لفئة تشخيصية.

- في بداية كل وحدة تشخيصية (باستثناء وحدة الاضطرابات الذهانية)، يتم عرض سؤال (أسئلة) الفحص المقابل للمعايير الرئيسية للاضطراب في مربع رمادي.
- في نهاية كل وحدة، يسمح مربع (مربعات) التشخيص للقائم على تطبيق المُقابلة بالإشارة إلى ما إذا كانت معايير التشخيص قد استوفيت أم لا.

المصطلحات المُتفق عليها:

- الجمل المكتوبة بـ « الخط العادي » يجب أن تقرأ تماما كما هو مكتوب للمريض من أجل توحيد تقييم معايير التشخيص.
- الجمل المكتوبة بـ « الخط الثقيل » لا ينبغي أن تقرأ للمريض. وهي تعليمات للقائم بتطبيق المقابلة للمساعدة في تسجيل التشخيص.
- الجمل المكتوبة و « تحتها خط » تشير إلى الإطار الزمني الذي يجري الاستفسار عنه. حيث ينبغي على الشخص الذي يجري المقابلة قراءتها كلما لزم الأمر. ويجب الالتفات فقط إلي الأعراض التي تحدث خلال الإطار الزمني المشار إليه أثناء تسجيل الإجابات.
- تُشير الإجابات التي يوجد فوقها سهم (➡) إلى أنه لم يتم استيفاء أحد المعايير اللازمة للتشخيص أو أنه لم يتم استيفاء التشخيص. في هذه الحالة، يجب أن يذهب الشخص الذي يُجري المقابلة إلى نهاية الوحدة وأن يتم وضع دائرة حول "لا" في جميع مربعات التشخيص الخاصة بالوحدة والانتقال إلى الوحدة التالية.
- عندما يتم فصل المصطلحات بشرطة مائلة (/) يجب أن يقرأ القائم بإجراء المقابلة فقط تلك الأعراض المعروف بوجودها في المريض (على سبيل المثال، الأسئلة J2b أو K6b).
- العبارات الموجودة بين الـ (أقواس) هي أمثلة إكلينيكية للأعراض. ويُمكن قراءتها للمريض لتوضيح الأسئلة.

تعليمات التقييم:

- يجب تقييم جميع الأسئلة. بحيث يتم التقييم على شمال كل سؤال عن طريق عمل دائرة حول نعم أو لا. يجب أن يعتمد القائم بإجراء المقابلة على حُكمه الإكلينيكي عند تسجيله إجابات الأسئلة. يجب أن يكون القائمين بإجراء المُقابلة حساسين لتنوع المعتقدات الثقافية أثناء تطبيقهم للأسئلة واختيارهم للإجابات. يجب أن يطلب القائم بإجراء المُقابلة أمثلة عند الضرورة، لضمان التسجيل الدقيق للإجابات. كما ينبغي تشجيع المريض على طلب توضيح بشأن أي مسألة غير واضحة تماما بالنسبة له.
- يجب أن يكون القائم بإجراء المقابلة متأكداً من أن المريض يراعى كل بُعد من أبعاد السؤال (على سبيل المثال، الإطار الزمني والتردد والشدة و / أو البدائل).
- الأعراض التي يُمكن تفسيرها بشكل أفضل بسبب عضوي أو عن طريق تعاطي الكحول أو المخدرات لا ينبغي أن يتم تسجيلها بإجابة إيجابية (نعم) في الـ M.I.N.I. لاسيما وأنه يوجد في الـ(M.I.N.I.) أسئلة تستفسر عن هذه المسائل.

© 2019 Charité Universitätsmedizin Berlin - Campus Benjamin Franklin. All rights reserved. Translated by Dana Churbaji, Shaymaa Hauary, Sara Mohamed & Carine Karnouk. Supervised by Dr. Malek Bajbouj.

A. نوبة اكتئاب كبرى
MAJOR DEPRESSIVE EPISODE

هذا السهم ← يعني انتقل إلى مربع التشخيص النهائي الخاص بالوحدة واختر (لا) في مربع التشخيص. انتقل بعدها إلى الوحدة التالية.

A1. a	هل سبق لك وأن شعرت بالاكتئاب أو الإحباط، أو هل شعرت بالحزن، أو شعرت بالفراغ أو اليأس، معظم اليوم، كل يوم تقريباً، لمدة أسبوعين؟	نعم	لا
	**إذا كانت الإجابة "لا"، فقم بتسجيل إجابة البند A1.b "لا":**<br>**إذا كانت الإجابة "نعم" اسأل التالي:**		
b	خلال الأسبوعين الماضيين، هل كنت مكتئبًا أو محبطاً، أو هل شعرت بالحزن، أو الفراغ أو اليأس، معظم اليوم، تقريبًا كل يوم؟	نعم	لا

A2. a	هل سبق لك وكنت أقل اهتماماً بمعظم الأشياء أو أقل قدرة على الاستمتاع بالأشياء التي اعتدت على الاستمتاع بها معظم الوقت، لمدة أسبوعين؟	نعم	لا
	**إذا كانت الإجابة "لا"، فقم بتسجيل إجابة البند A2.b "لا":**<br>**إذا كانت الإجابة "نعم" اسأل التالي:**		
b	خلال الأسبوعين الماضيين، هل كنت أقل اهتماماً بمعظم الأشياء أو أقل قدرة على الاستمتاع بالأشياء التي اعتدت على الاستمتاع بها, معظم الوقت؟	نعم	لا

**هل أجبت بـ "نعم" على البند A1.a أو البند A2.a؟**	نعم	لا ←

A3	**إذا كانت الإجابة على البند A1.b أو البند A2.b "نعم", استعلم عن النوبة الحالية والنوبة ذات الأعراض الأكثر شدّة في الماضي، وإلا ..**<br>**إذا كانت إجابة كلاً من البند A1.b و البند A2.b "لا" استعلم فقط عن النوبة ذات الأعراض الأكثر شدة في الماضي.**					
	خلال فترة الأسبوعين، عندما شعرت بالاكتئاب أو بعدم الاهتمام:	**الأسبوعين الماضيين**		**النوبة الماضية**		
a	هل انخفضت أو ازدادت شهيتك كل يوم تقريبًا؟ هل انخفض أو ازداد وزنك دون قصدٍ منك (على سبيل المثال، ±5% من وزن الجسم، أو ما يعادل 8 أرطال أو ±3,5 كجم في شهرٍ واحد لشخص يبلغ وزنه 160 رطل/ 70 كجم)؟	**نعم**	**لا**	**نعم**	**لا**	
b	هل واجهتك صعوبات في النوم، كل ليلة تقريباً؟ (صعوبة في الخلود إلى النوم، أو الاستيقاظ في منتصف الليل, أو الاستيقاظ في وقت مبكر جداً, أو النوم بشكل مفرط)	**نعم**	**لا**	**نعم**	**لا**	
c	هل تحدثت أو تحركت بطريقة أبطأ من المعتاد أو كنت عصبي، مُتململ أو تواجه صعوبة في البقاء ساكناً، كل يوم تقريباً؟ هل لاحظ أحدهم ذلك؟	**نعم**	**لا**	**نعم**	**لا**	
d	هل شعرت بالتعب وبفقدان الطاقة\الحيوية، كل يوم تقريباً؟	**نعم**	**لا**	**نعم**	**لا**	

© 2019 Charité Universitätsmedizin Berlin - Campus Benjamin Franklin. All rights reserved. Translated by Dana Churbaji, Shaymaa Hauary, Sara Mohamed & Carine Karnouk. Supervised by Dr. Malek Bajbouj.

	هل شعرت أنك بلا قيمة أو رافقك شعور بالذنب، كل يوم تقريباً؟	نعم	لا	نعم	لا
e	**إذا كانت الإجابة "نعم"، اسأل عن أمثلة. ابحث عن *ضلالات (Delusions) الفشل أو عدم الكفاءة أو الخراب أو الشعور بالذنب أو الحاجة إلى العقاب أو ضلالات المرض أو الوفاة أو الضلالات العدمية (أن الحياة بلا معنى) أو الجسدية. على الأمثلة أن تتسق مع فكرة ضلالية.**<br><br>***الضلالات هي معتقدات/معتقد خاطئ يؤمن به المريض على الرغم من تناقضه مع الواقع أو تناقضه مع الحجة المنطقية، وتعتبر الضلالات عادةً كأحد أعراض الاضطراب العقلي (الذهاني).**	نعم	لا	نعم	لا
f	هل واجهتك صعوبات في التركيز، التفكير أو في اتخاذ القرارات، كل يوم تقريباً؟	نعم	لا	نعم	لا
g	هل فكرت بشكل متكرر في الموت (**الخوف من الموت لا يُحتسب هنا**)، أو كانت لديك أي أفكار بقتل نفسك، أو كانت لديك أي نية أو خطة لقتل نفسك؟ هل حاولت الانتحار؟ إذا كانت الإجابة بنعم على أياً من هذه الأسئلة، ضع علامة نعم	نعم	لا	نعم	لا
A4	هل سببت لك هذه الأعراض بمعاناة ملحوظة (كبيرة) أو سببت لك مشاكل في المنزل أو العمل أو المدرسة أو في علاقاتك الاجتماعية أو بطريقة أخرى ذات أهمية، وهل تغيّر أداؤك في أحد هذه الجوانب المذكورة عن السابق؟	نعم	لا	نعم	لا
A5	بين نوبة اكتئاب وأخرى، هل سبق لك وأمضيت مدّة لا تقل عن شهرين، لم تشعر فيها بأي اكتئاب أو فقدان اهتمام؟	نعم	لا	نعم	لا

	**هل قمت بتسجيل 5 إجابات أو أكثر بـ "نعم" في أسئلة البنود (A1-A3) وهل تم تسجيل إجابة "نعم" على البند A4 في تلك الفترة الزمنية؟**	نعم	لا	نعم	لا
	**و هل تم تسجيل إجابة "نعم" على البند الخاص بـ "استبعاد السبب العضوي" التابع للملخص O2**	نعم	لا	نعم	لا
	**حدد إذا كانت النوبة حالية و(/أو) ماضية.**<br>**إذا كانت إجابة A5 "نعم" فقم بتسجيل "نعم" عند نوبة متكررة الحدوث " Recurrent Episode"**				

نوبة اكتئاب كبرى<br>Major Depressive Episode	
حالي	☐
ماضي	☐
متكررة الحدوث	☐

A6	كم عدد نوبات الاكتئاب التي مررت بها في حياتك؟<br>يجب أن يكون هناك فاصل زمني لا يقل عن شهرين بين كل نوبة وأخرى بدون أي اكتئاب ملحوظ	______

© 2019 Charité Universitätsmedizin Berlin - Campus Benjamin Franklin. All rights reserved. Translated by Dana Churbaji, Shaymaa Hauary, Sara Mohamed & Carine Karnouk. Supervised by Dr. Malek Bajbouj.

B. الانتحار
SUICIDALITY

	<u>في الشهر الماضي:</u>			النقاط
B1	هل تعرضت لأي حادث؟ يشمل ذلك تناولك كمية كبيرة من أدويتك دون قصد (عن طريق الخطأ)	نعم	لا	0
	**إذا كانت الإجابة على البند B1 "لا", انتقل إلى البندB2**<br>**إذا كانت الإجابة "نعم" اسأل B1.a**			
B1. a	هل خطت أو قصدت إيذاء نفسك في أي حادث إما عن طريق عدم تجنب المخاطر عند حدوثها أو عن طريق التسبب بالحادث عن قصد؟	نعم	لا	0
	**إذا كانت الإجابة على البند B1.a "لا", انتقل إلى البند B2**<br>**إذا كانت الإجابة "نعم" اسأل B1b**			
B1. b	هل تعمدت الموت كنتيجة لأي حادث؟	نعم	لا	0
B2	هل فكرت (حتى للحظات) بأنك ستكون أفضل حالاً لو مُت أو تمنيت أن تكون ميتاً أو أنك بحاجة للموت؟	نعم	لا	1
B3	هل فكرت (حتى للحظات) بإيذاء، إصابة أو جرح نفسك؟<br>- مع وجود (ولو بعض) التعمد أو الوعي بأنك قد تموت نتيجةً لذلك.<br>- أو التفكير بالانتحار (أي قتل نفسك)؟	نعم	لا	6
	**إذا كانت إجابة البند B3 + B2 بـ "لا"، فانتقل إلى B4**<br>**عدا ذلك اسأل عن التالي:**			

التكرار:		الشدة:	
أحياناً	☐	خفيفة	☐
غالباً	☐	متوسطة	☐
كثيراً	☐	شديدة	☐

B4	هل سمعت صوتاً أو أصواتاً تقول لك أن تقتل نفسك أو راودك حلم له علاقة بالانتحار؟	نعم	لا	4
	**إذا كانت الإجابة "نعم", اختر أحد هذين الخيارين أو كليهما**			
	هل كان صوتا واحداً أم عدة أصوات؟ ☐		هل كان هذا حلما؟ ☐	

B5	هل فكرت بطريقة معينة للانتحار (كيف)؟	نعم	لا	8
B6	هل فكرت بوسيلة انتحار معينة (ما هي)؟	نعم	لا	8
B7	هل فكرت بمكان معين لمحاولة الانتحار (أين)؟	نعم	لا	8
B8	هل فكرت بتاريخ أو إطار زمني معين للانتحار (متى)؟	نعم	لا	8
B9	هل فكرت في أي مهمة ترغب بالقيام بها قبل محاولتك قتل نفسك؟ (على سبيل المثال: كتابة رسالة انتحار)	نعم	لا	8
B10	هل كنت تنوي تنفيذ الأفكار المتعلقة بقتل نفسك؟	نعم	لا	8
	**إذا كانت الإجابة "نعم", اختر أحد هذين الخيارين أو كليهما**			
	هل كنت تنوي تنفيذ مخططاتك في ذلك الوقت؟ ☐		هل كنت تنوي تنفيذ مخططاتك في وقت ما في المستقبل؟ ☐	

B11	هل كنت تنوي الموت نتيجة لتصرف انتحاري؟	نعم	لا	8
	**إذا كانت الإجابة "نعم", اختر أحد هذين الخيارين أو كليهما**			
	هل كنت تنوي الموت في ذلك الوقت؟ ☐		هل كنت تنوي الموت في وقت ما في المستقبل؟ ☐	

© 2019 Charité Universitätsmedizin Berlin - Campus Benjamin Franklin. All rights reserved. Translated by Dana Churbaji, Shaymaa Hauary, Sara Mohamed & Carine Karnouk. Supervised by Dr. Malek Bajbouj.

B12	هل شعرت بحاجة أو بدافع (رغبة قوية ومُلحة) لقتل نفسك أو التخطيط لقتل نفسك عاجلاً دون تأجيل؟	نعم	لا	8
	**إذا كانت الإجابة "نعم", اختر أحد هذين الخيارين أو كليهما**			
	هل ارتبطت هذه الرغبة/الدافع بقتلك لنفسك؟	☐	هل ارتبطت هذه الرغبة/الدافع بالتخطيط لقتل نفسك؟	☐
	**إذا كانت الإجابة "نعم", اختر أحد هذين الخيارين أو كليهما**			
	هل كانت هذه الرغبة/الدافع بشكل عام غير ناتجة عن حافز أو سبب مباشر؟ (ليست ناتجة عن حدث أو مشاعر معيّنة)	☐	هل كانت هذه الرغبة/الدافع ناتجة عن حافز أو سبب مباشر؟ (نتيجة حدث معين أو مشاعر معينة)	☐

**عند تقييم لكون هذا الدافع أو الرغبة المُلحة غير ناتجة بشكل عام عن حافز أو سبب مباشر، اسأل:** "قبل ٥ دقائق من هذا الدافع، هل كان بإمكانك التنبؤ به"؟<br><br>**إذا كان الإجابة على البند B12 "لا"، انتقل إلى البند B14.**	نعم	لا

B13	هل تواجه صعوبة في مقاومة هذه الدوافع المُلحة/القوية؟	نعم	لا	8
B14	هل اتخذت أي خطوات عمليّة للتحضير لمحاولة انتحار توقعت أو قصدت فيها الموت (بما في ذلك أي فعل مقصود أو غير مقصود دفعك للقيام بمحاولة انتحار)؟<br><br>يتضمن ذلك الأوقات التي كنت ستقتل فيها نفسك، ولكن تمت مقاطعتك أو توقفت، قبل إيذاء نفسك.	نعم	لا	
B14.a	هل اتخذت خطوات عمليّة للتحضير لقتل نفسك، لكنك لم تبدأ بمحاولة الانتحار؟	نعم	لا	9
B14.b	هل اتخذت خطوات عمليّة للتحضير لقتل نفسك، لكنك بعدها توقفت مباشرةً قبل إلحاق الأذى بنفسك ("لم تكتمل المحاولة")	نعم	لا	10
B14.c	هل اتخذت خطوات عمليّة للتحضير لقتل نفسك، ولكن شخصاً ما أو شيء ما أوقفك مباشرةً قبل إلحاق الأذى بنفسك ("تم مقاطعتك أثناء المحاولة")	نعم	لا	11
B15	هل جرحت/أذيت نفسك عمداً، لكن دون أن تنوي قتل نفسك؟	نعم	لا	0
B16	هل حاولت الانتحار (لقتل نفسك)؟<br>إذا كانت الإجابة على البند B16 "لا"، انتقل إلى البند B17.	نعم	لا	
B16.a	هل بدأت بمحاولة انتحار (لقتل نفسك)، ولكنك قررت التوقف ولم تُكمل المحاولة؟	نعم	لا	12
B16.b	هل بدأت بمحاولة انتحار (لقتل نفسك)، ولكن تمت مُقاطعتك ولم تُكمل المحاولة؟	نعم	لا	13
B16.c	هل قمت بمحاولة انتحار (لقتل نفسك)، تماماً كما كان مخططاً لها؟ محاولة الانتحار تعني أنك قمت بفعل معين كان يمكن أن يلحق بك الأذى، مع وجود ولو نية بسيطة للموت.<br>**إذا كانت الإجابة على البند B16c "لا"، فانتقل إلى البند B17**	نعم	لا	14
	تمنيت أن يتم إنقاذك/أن تنجو	☐	توقعت/تمنيت الموت	☐

B17	الوقت الذي تقضيه في اليوم مع دوافع ملحة، أفكار أو أفعال انتحار:		
	الوقت المعتاد الذي تقضيه في اليوم	ساعة:	دقيقة:
	أقل قدر من الوقت الذي تقضيه في اليوم	ساعة:	دقيقة:
	أكبر قدر من الوقت الذي تقضية في اليوم	ساعة:	دقيقة:

B18	على مدار حياتك هل سبق لك أن حاولت الانتحار (حاولت أن تقتل نفسك)؟	نعم	لا	4
	**إذا كانت الإجابة "نعم"، كم مرة؟ ____________**			

© 2019 Charité Universitätsmedizin Berlin - Campus Benjamin Franklin. All rights reserved. Translated by Dana Churbaji, Shaymaa Hauary, Sara Mohamed & Carine Karnouk. Supervised by Dr. Malek Bajbouj.

	**إذا كانت الإجابة "نعم"، متى كانت آخر محاولة انتحار؟**					
	مرحلة حالية: خلال الـ ١٢ شهراً الماضية	☐	مرحلة تعافي مُبكر: ما بين الـ ١٢ والـ٢٤ شهراً الماضية	☐	مرحلة تعافي: منذ أكثر من ٢٤ شهر	☐
	**"محاولة الانتحار هي أي سلوك مُضر بالنفس، مع توافر ولو شيء من التعمد البسيط (> 0) للموت كنتيجة لهذا الفعل. قد يكون الدليل على أن الفرد يعتزم قتل نفسه (بدرجة معينة على الأقل) على شكل قول صريح أو يمكن الاستدلال عليه من السلوك أو الموقف. على سبيل المثال، يعّرف الفعل على أنه محاولة انتحار إذا لم يكن حادثاً بشكل واضح أو إذا ظن الفرد أن الفعل قد يكون قاتلاً، على الرغم من إنكاره النية لذلك."**<br><br>(FDA Guidance for Industry Suicidal Ideation and Behavior Document 2012 and C-CASA definition). Posner K et al. Am J Psychiatry 2007; 164 (7): 1035-1043 & http://www.fda.gov/Drugs/GuidanceComplianceRegulatoryInformation/Guidances/default.htm/					
B19	ما هي احتمالية قيامك بمحاولة قتل نفسك في غضون الأشهر الثلاثة القادمة؟ 0-100% ________________<br><br>**إذا كان الاحتمال أكبر من الصفر، سجّل "نعم" في البند B19**					13

**هل قُمت بتسجيل إجابة "نعم" في واحد من البنود السابقة على الأقل؟ (باستثناء B1)**	نعم	لا
**إذا كانت الإجابة "نعم"، احسب مجموع النقاط للبنود (B1-B19) التي تم الإجابة عليها بـ "نعم".**<br>**حدد بناءً على عدد النقاط فئة خطر الانتحار كما هو موضح في مربع التشخيص:**		
**حدد ما إذا كان سلوك الانتحار حالي (حدث في الشهر الماضي)**<br>**أو مزمن مدى الحياة، أو كليهما عن طريق تحديد الإجابة المناسبة في مربع التشخيص (أو ترك أحدهما أو كليهما دون علامة).**		

**حالي: إذا تمت الإجابة بـ "نعم" على كل البنود من (B1.a - B16.c باستثناء B15)**<br>**أو في حال وجود أي وقت مستغرق في البند B17**	**محاولات مزمنة مدى الحياة: إذا تمت الإجابة بـ "نعم" على البند B18**	**احتمال وارد في المستقبل القريب: إذا تمت الإجابة بـ "نعم" على البند B19**	

**يمكنك كتابة أي تعليقات إضافية حول تقييمك للمريض فيما يخص سلوك الانتحار الحالي أو في المستقبل القريب في المكان/الفراغ التالي:**

الانتحار<br>SUICIDALITY			
ضعيف = 1 - 8 نقطة	☐	حالي	☐
متوسط = 9 - 16 نقطة	☐	محاولات مزمنة مدى الحياة	☐
مرتفع = فوق 17 نقطة	☐	احتمال وارد في المستقبل القريب	☐

© 2019 Charité Universitätsmedizin Berlin - Campus Benjamin Franklin. All rights reserved. Translated by Dana Churbaji, Shaymaa Hauary, Sara Mohamed & Carine Karnouk. Supervised by Dr. Malek Bajbouj.

**هل تم تسجيل إجابة "نعم" على البند B18؟**	نعم	لا
**والإجابة بـ "نعم" على السؤال التالي؟**<br>**هل تم البدء بمحاولة الانتحار في حين لم يكن الشخص في حالة من التشوش والهذيان؟**	نعم	لا
**والإجابة بـ "نعم" على السؤال التالي؟**<br>**هل تم القيام بفعل الانتحار دون وجود سبب سياسي أو ديني؟**	نعم	لا
**إذا كانت الإجابة "نعم"، حدد ما إذا كان الاضطراب حالي (Current) أم لا،**<br>**أو ما إذا كان في مرحلة تعافي مبكر "Early Remission" أو في مرحلة تعافي "Remission"**<br><br>**(ملحوظة: Remission - تعني فترة زمنية يكون فيها المرض أو الأعراض أقل حدة، وتبدء معايير التشخيص الخاصة بالمرض في الانخفاض بشكل كبير بحيث لا تستوفي أعراض المريض في هذه المرحلة معايير التشخيص الخاصة بالمرض)**		

اضطراب السلوك الانتحاري<br>SUICIDE BEHAVIOR DISORDER	
حالي	☐
في مرحلة تعافي مبكر	☐
في مرحلة تعافي	☐

© 2019 Charité Universitätsmedizin Berlin - Campus Benjamin Franklin. All rights reserved. Translated by Dana Churbaji, Shaymaa Hauary, Sara Mohamed & Carine Karnouk. Supervised by Dr. Malek Bajbouj.

نوبات الهوس والهوس الخفيف .C
MANIC AND HYPOMANIC EPISODES

هذا السهم ← يعني انتقل إلى مربع التشخيص النهائي الخاص بالوحدة واختر (لا) في مربع التشخيص. انتقل بعدها إلى الوحدة التالية.

	هل لديك أي تاريخ عائلي مع مرض الهوس/الاكتئاب/اضطراب ثنائي القطب، أو هل كان أحد أفراد أسرتك يعاني من تقلبات مزاجية وكان يتناول دواء مثل الليثيوم، فالبروات الصوديوم (Depakote) أو لاموتريجين (Lamictal)؟	نعم	لا
	**هذا السؤال ليس معيارًا لتشخيص اضطراب ثنائي القطب، ولكن الغرض منه زيادة يقظة القائم بإجراء المقابلة بشأن خطر الإصابة أو قابلية الإصابة باضطراب ثنائي القطب.**		
	**إذا كانت الإجابة "نعم" يرجى تحديد من:**		
C1. a	هل سبق لك أن مررت بفترة من الوقت كنت تشعر فيها بأن لديك حالة من " النشوة " أو " الابتهاج " أو "النشاط الزائد والاهتياج" أو أنك نشيط جداً ومليء بالطاقة أو لديك حيوية مفرطة لدرجة سببت لك مشكلة نتيجة لذلك، أو أن أشخاصًا آخرين اعتقدوا أنك لست على ما يرام أو لست الشخص الذي اعتادوا عليه؟ (لا تضع في الاعتبار الأوقات التي كنت فيها تحت تأثير الكحول أو المواد المخدرة)	نعم	لا
	**إذا كان المريض مُحتاراً أو لا يفهم ماذا تعني بـ " النشوة" أو "الابتهاج" أو "النشاط الزائد والاهتياج"، أوضح ماذا تعني بذلك كما يلي: أعني بذلك** وجود مزاج مُبتهج، وازدياد في الطاقة والنشاط، والحاجة إلى قدر قليل من النوم، وتسارع الأفكار؛ وزيادة في الإنتاجية، والحافز، والإبداع، إضافة إلى السلوك المتهور. التحدث عبر الهاتف بشكل مفرط، أو العمل بشكل مفرط أو إنفاق المزيد من المال.		
	**إذا كانت الإجابة "لا"، فقم بتسجيل إجابة "لا" في البند C1.b**<br>**أما إذا كانت الإجابة "نعم" اسأل التالي:**		
C1. b	هل تشعر في الوقت الحالي بالـ "بالابتهاج والنشوة" أو "الاستثارة" أو "النشاط الزائد والاهتياج" أو أنك مليء بالطاقة؟	نعم	لا
C2. a	هل سبق لك أن كنت في حالة من التهيُج المستمر، لعدة أيام، بحيث دخلت نتيجة لذلك في منازعات أو مشاحنات لفظية أو جسدية، أو صرخت بوجه أشخاص من خارج أفراد أسرتك؟ هل لاحظت أنت أو غيرك أن غضبك أو رد فعلك كان مبالغ فيه، مقارنةً بالأشخاص الآخرين، حتى في المواقف التي شعرت فيها أن تصرفاتك مبررة؟	نعم	لا
	**إذا كانت الإجابة "لا"، فقم بتسجيل إجابة "لا" في البند C2.b**<br>**أما إذا كانت الإجابة "نعم" اسأل التالي:**		
C2. b	هل تشعر في الوقت الحالي أنك متهيج أو منفعل بشكل دائم؟	نعم	لا ←
	**هل تم تسجيل إجابة "نعم" في البند C1.a أو C2.a؟**	نعم	لا

© 2019 Charité Universitätsmedizin Berlin - Campus Benjamin Franklin. All rights reserved. Translated by Dana Churbaji, Shaymaa Hauary, Sara Mohamed & Carine Karnouk. Supervised by Dr. Malek Bajbouj.

C3

إذا تم تسجيل إجابة "نعم" على البند C1.a أو C2.a, استفسر عن النوبة الحالية أولاً، ثم استفسر عن النوبة الماضية الأكثر شدّة، عدا ذلك..

إذا تم تسجيل إجابة "لا" على البند C1.a أو C2.a, استفسر فقط عن النوبة الماضية الأكثر شدّة.

عند الاستفسار عن النوبة الحالية، قم بتقديم كل سؤال على النحو التالي:

خلال الأيام القليلة الماضية، بما في ذلك اليوم، عندما شعرت فيها بالابتهاج ، أو أنك مُفعم بالطاقة والحيوية ، أو الاهتياج والانفعال، هل:

عند الاستفسار عن النوبة الماضية, قم بتقديم السؤال على النحو التالي:

خلال الأيام القليلة الماضية التي كنت تشعر فيها بأنك في أقصى حالات الابتهاج والطاقة وبأنك منفعل إلى أقصى درجة, هل:

		الأسبوعين الماضيين		النوبة الماضية	
C3.a	هل شعرت أن باستطاعتك القيام بأمور لا يستطيع الآخرون القيام بها؟ أو بأنك شخص مهم للغاية؟<br>إذا تم تسجيل إجابة "نعم", اسأل عن أمثلة (على الأمثلة أن تتسق مع فكرة ضلالية)	نعم	لا	نعم	لا
C3.b	تحتاج إلى قدر أقل من النوم (على سبيل المثال, تشعر بالراحة بعد ساعات نوم قليلة)	نعم	لا	نعم	لا
C3.c	هل كنت تتحدث كثيرًا دون توقف، أو شعرت بضغط (دافع؟) وأن هناك ما يدفعك لمواصلة الحديث دون توقف.	نعم	لا	نعم	لا
C3.d	هل لاحظت أن أفكارك تتابع بسرعة كبيرة أو تتسارع مع بعضها البعض أو تتسابق أو أنك تنتقل بسرعة كبيرة من موضوع إلى آخر؟	نعم	لا	نعم	لا
C3.e	هل أصبحت تتشتت بسهولة لدرجة أن أي تشويش بسيط يمكن أن يُشتت انتباهك؟	نعم	لا	نعم	لا
C3.f	هل كانت لديك زيادة كبيرة في النشاط والدافع ، في العمل، أو في المدرسة أو اجتماعيا أو جنسيا أو هل أصبحت متململ جسديا أو نفسيا ولا يمكنك أن تهدأ؟ قد تكون هذه الزيادة في النشاط مع أو بدون هدف أو غرض معين.	نعم	لا	نعم	لا
C3.g	هل كانت لديك رغبة مُلحة للانخراط في أنشطة مُمتعة لدرجة أنك تجاهلت المخاطر أو العواقب المترتبة عليها (على سبيل المثال: الإسراف في صرف النقود, أو القيادة بشكل متهور, أو التصرفات الجنسية الغير لائقة)؟	نعم	لا	نعم	لا
C3 SUMMARY	**عند تقييمك للنوبة الحالية:**<br>**إذا كانت الإجابة على البند C1.b "لا"، فهل تم تسجيل 4 إجابات أو أكثر من أسئلة البند C3 بما في ذلك C3.f بـ "نعم"؟**<br>**إذا كانت الإجابة على C1.b "نعم"، فهل تم تسجيل 3 إجابات أو أكثر من أسئلة البند C3 بما في ذلك C3.f بـ "نعم"؟**	نعم	لا		
	**عند تقييمك للنوبة الماضية:**<br>**إذا كانت الإجابة على البند C1.b "لا"، فهل تم تسجيل 4 إجابات أو أكثر من أسئلة البند C3 بما في ذلك C3.f بـ "نعم"؟**<br>**إذا كانت الإجابة على C1.b "نعم"، فهل تم تسجيل 3 إجابات أو أكثر من أسئلة البند C3 بما في ذلك C3.f بـ "نعم"؟**			نعم	لا

© 2019 Charité Universitätsmedizin Berlin - Campus Benjamin Franklin. All rights reserved. Translated by Dana Churbaji, Shaymaa Hauary, Sara Mohamed & Carine Karnouk. Supervised by Dr. Malek Bajbouj.

	**سجّل إجابة "نعم" في حال حدوث الـ ٣ أو الـ ٤ أعراض المذكورة أعلاه خلال فترة زمنية واحدة.**	
	**القاعدة: الابتهاج (النشوة)/ المزاج المرتفع, لا يتطلب سوا ٣ من أعراض البند, C3 بينما يتطلب الانفعال (الاهتياج) وجود ٤ من أعراض البند C3.**	

C4	ما هي أطول فترة استمرت فيها هذه الأعراض (معظم اليوم كل يوم تقريباً)؟				
	**قم بتقييم المدة الزمنية منذ بداية إلى نهاية الأعراض، وليس فقط فترة الذروة (عندما كانت الأعراض شديدة وواضحة).**				
	**a) ثلاثة أيام متتالية أو أقل**	☐		☐	
	**b) أربعة أو خمسة أو ستة أيام متتالية أو أكثر**	☐		☐	
	**c) سبع أيام متتالية أو أكثر**	☐		☐	
C5	هل أقمت في المستشفى نتيجة لهذه المشاكل؟**إذا تم تسجيل إجابة "نعم", اختر الإجابة "نعم" في نوبة الهوس لتلك الفترة الزمنية وانتقل إلى البند C7.**	نعم	لا	نعم	لا
	**إذا تم تسجيل إجابة "نعم", اختر الإجابة "نعم" في نوبة الهوس لتلك الفترة الزمنية وانتقل إلى البند C7.**				
C6	هل تسببت هذه الأعراض في حدوث مشاكل ملحوظة في المنزل أو في العمل أو اجتماعيًا في علاقاتك بالآخرين أو في المدرسة أو أثرت عليك بطريقة أخرى ذات أهمية؟	نعم	لا	نعم	لا
C7	هل كانت هذه الأعراض مرتبطة بتغيير ملحوظ في طريقة تعاملك وتصرفك السابقة بحيث اختلفت عن طريقتك المعتادة؟	نعم	لا	نعم	لا

	**هل تم تسجيل إجابة "نعم" في البنود التالية: ملخص البند C3 و C7 و (C4.c أو C5 أو C6 أو أي من الملامح الذهانية الموجودة من البند K1 حتى بند K8)؟**	نعم	لا
	**و هل تم تسجيل إجابة"نعم" على البند الخاص بـ "استبعاد السبب العضوي" التابع للملخص O2؟**		
	**إذا كانت الإجابة "نعم" حدد إذا كانت النوبة حالية و(/أو) ماضية**		

نوبة هوس<br>MANIC EPISODE	
حالي	☐
ماضي	☐

© 2019 Charité Universitätsmedizin Berlin - Campus Benjamin Franklin. All rights reserved. Translated by Dana Churbaji, Shaymaa Hauary, Sara Mohamed & Carine Karnouk. Supervised by Dr. Malek Bajbouj.

	هل تم تسجيل إجابة "نعم" في ملخص البند C3<br>و "لا" في البنودC5; C6<br>و"نعم" في البندC7<br>وهل تم تسجيل إجابة "نعم" على C4.c أو C4.b؟	نعم	لا
	و هل تم تسجيل إجابة "نعم" على البند الخاص بـ "استبعاد السبب العضوي" التابع للملخص O2؟		
	وهل تم تسجيل إجابة "لا" على جميع الملامح الذهانية "Psychotic Features" الموجودة في البنود من K1 لـ K8 ؟		
	إذا كانت الإجابة "نعم" حدد إذا كانت النوبة حالية و(/أو) ماضية		
	في حال تسجيل إجابة "نعم" عند نوبة الهوس الحالية, قم بتسجيل "لا" عند نوبة الهوس الخفيف.		
	إذا كانت الإجابة "نعم" على نوبة الهوس الماضية ، فقم بتدوين نوبة الهوس الخفيف بـ "لم يتم استكشافها" في مربع التشخيص.		

نوبة هوس خفيف<br>HYPOMANIC EPISODE				
حالية		ماضية		
نعم	لا	نعم	لا	لم يتم استكشافها

	هل تم تسجيل إجابة "نعم" على ملخص البند C3 و C4.a و تم تسجيل "لا" عند البند C5؟	نعم	لا
	حدد إذا كانت النوبة حالية و / أو ماضية		
	إذا تم تسجيل إجابة "نعم" على نوبة الهوس الحالية أو نوبة الهوس الخفيفة الحالية, فقم بتسجيل "لا" في مربع تشخيص أعراض الهوس الخفيف.		
	إذا تم تسجيل "نعم" على نوبة الهوس السابقة أو نوبة الهوس الخفيف السابقة, فقم بتسجيل "لم يتم استكشافها" عند أعراض الهوس الخفيف السابقة		

أعراض الهوس الخفيف<br>HYPOMANIC SYMPTOMS				
حالية		ماضية		
نعم	لا	نعم	لا	لم يتم استكشافها

C8. a	إذا كانت نوبة الهوس إيجابية في مربع التشخيص، سواء كانت حالية أو ماضية، اسأل التالي:	نعم	لا
	على مدار حياتك, هل كان لديك 2 أو أكثر من هذه النوبات (الهوس) وكانت تستمر لـ 7 أيام أو أكثر (C4.c)؟		

© 2019 Charité Universitätsmedizin Berlin - Campus Benjamin Franklin. All rights reserved. Translated by Dana Churbaji, Shaymaa Hauary, Sara Mohamed & Carine Karnouk. Supervised by Dr. Malek Bajbouj.

	**(بما في ذلك النوبة الحالية إذا كانت موجودة)**		
C8. b	**إذا كانت نوبة الهوس أو الهوس الخفيف إيجابية في مربع التشخيص, سواء حالية أو ماضية, اسأل التالي:**<br>على مدار حياتك, هل كان لديك 2 أو أكثر من هذه النوبات (الهوس الخفيف) وكانت تستمر لـ ٤ أيام أو أكثر(C4.b)؟<br>**(بما في ذلك النوبة الحالية)**	نعم	لا
C8. c	**إذا كان مربع تشخيص أعراض الهوس الخفيف السابق إيجابي, اسأل التالي:**<br>هل أصابتك أعراض الهوس الخفيف هذه مرتين أو أكثر على مدار حياتك واستمرت من يوم إلى ثلاثة أيام فقط (C4a) ، (بما في ذلك النوبة الحالية إن وجدت)؟	نعم	لا

© 2019 Charité Universitätsmedizin Berlin - Campus Benjamin Franklin. All rights reserved. Translated by Dana Churbaji, Shaymaa Hauary, Sara Mohamed & Carine Karnouk. Supervised by Dr. Malek Bajbouj.

D. اضطراب الهلع
PANIC DISORDER

هذا السهم ← يعني انتقل إلى مربع التشخيص النهائي الخاص بالوحدة واختر (لا) في مربع التشخيص. انتقل بعدها إلى الوحدة التالية.

D1. a	هل تعرضت, في أكثر من مناسبة, لنوبات شعرت فيها **فجأة** بالقلق أو الخوف أو عدم الارتياح, حتى في مواقف لا يشعر فيها معظم الناس بهذه الطريقة؟	نعم	لا ←
D1. b	هل تصاعدت هذه النوبات حتي وصلت لذروتها في خلال 10 دقائق من بدايتها؟	نعم	لا ←
D2	في أي وقت في الماضي, هل حدثت أي من تلك النوبات بشكل مفاجئ, غير متوقع أو بدون سبب يذكر؟	نعم	لا ←
D3	هل سبق أن كانت أحد هذه النوبات متبوعة, بـ شهر أو أكثر, من الخوف المستمر من عودة نوبة مشابهة, أو من القلق اتجاه عواقب هذه النوبات, أو هل قمت بتغيير ملحوظ في تصرفاتك نتيجة لهذه النوبات (كتجنب المواقف الغير مألوفة, أو تجنب مغادرة المنزل أو التسوق بمفردك, أو القيام بتصرفات لتجنب التعرض لنوبة هلع, أو زيارة الطبيب أو غرفة الطوارئ بشكل متكرر)؟	نعم	لا
D4	خلال أشد نوبة يمكنك تذكرها:		
a.	هل شعرت بتسارع، غياب أو خفقان ضربات قلبك, أو عدم انتظامها؟	نعم	لا
b.	هل كانت يداك متعرقه أو رطبة؟	نعم	لا
c.	هل كنت ترتجف او ترتعش؟	نعم	لا
d.	هل كان لديك ضيق في التنفس أو صعوبة في التنفس أو إحساس بالاختناق؟	نعم	لا
e.	هل كان لديك إحساس بالاختتناق أو بغصة في حلقك؟	نعم	لا
f.	هل كان لديك آلام, ضغط, ضيق أو عدم راحة في الصدر؟	نعم	لا
g.	هل كنت تعاني من الغثيان, الإسهال المفاجئ أو مشاكل في المعدة؟	نعم	لا
h.	هل شعرت بالدوار أو اختلال التوازن أو شعرت أنك على وشك السقوط أو الإغماء؟	نعم	لا
i.	هل شعرت بهبات ساخنة (شعور مفاجئ بالحرارة والسخونة) أو قشعريرة؟	نعم	لا
j.	هل شعرت وخز (تنميل) أو خدر في أجزاء جسدك؟	نعم	لا
k.	هل شعرت أن الأشياء من حولك كانت غريبة أو غير واقعية أو منفصلة أو غير مألوفة، أو هل شعرت بأنك انعزلت أو انفصلت عن جسدك بالكامل أو عن جزء منه؟	نعم	لا
l.	هل كنت تخشى الإصابة بالاختلال أو فكرة فقدان السيطرة؟	نعم	لا
m.	هل كنت خائف من كونك تحتضر (تموت)؟	نعم	لا
D5	**هل تم تسجيل إجابة "نعم" عند البند D3 (نعم)**<br>**وعلى ٤ أو أكثر من أسئلة البند D4؟**	***نعم***	لا ←
نعم= اضطراب هلع مزمن مدى الحياة<br>***PANIC DISORDER LIFETIME***			

D6	<u>في الشهر الماضي</u>، هل صاحبك خوف مستمر من عودة النوبات مرة أخرى، أو قلق بشأن عواقب تلك النوبات, أو هل غيرت سلوكك بأي شكل من الأشكال بسبب تلك النوبات؟	***نعم***	لا
نعم= اضطراب هلع حالي<br>***PANIC DISORDER CURRENT***			

© 2019 Charité Universitätsmedizin Berlin - Campus Benjamin Franklin. All rights reserved. Translated by Dana Churbaji, Shaymaa Hauary, Sara Mohamed & Carine Karnouk. Supervised by Dr. Malek Bajbouj.

لا	نعم	**هل تم تسجيل إجابة "نعم" على البند D5 و D6؟**	
		**هل تم تسجيل إجابة "نعم" على البند الخاص بـ "استبعاد السبب العضوي" التابع للملخص O2؟**	
		**حدد ما إذا كانت النوبة حالية و / أو مزمنة مدى الحياة**	
اضطراب الهلع<br>PANIC DISORDER			
☐	حالي		
☐	مزمن مدى الحياة		

© 2019 Charité Universitätsmedizin Berlin - Campus Benjamin Franklin. All rights reserved. Translated by Dana Churbaji, Shaymaa Hauary, Sara Mohamed & Carine Karnouk. Supervised by Dr. Malek Bajbouj.

E. رهاب الأماكن المكشوفة/العامة
AGORAPHOBIA

هذا السهم ← يعني انتقل إلى مربع التشخيص النهائي الخاص بالوحدة واختر (لا) في مربع التشخيص. انتقل بعدها إلى الوحدة التالية.

E1	هل تشعر بالقلق أو عدم الارتياح في الأماكن أو المواقف التي قد لا تتاح فيها المساعدة أو قد يكون الهروب منها صعبًا إذا أصبت بنوبة هلع أو أعراض شبيهة بالهلع أو الخجل ، مثل: التواجد وسط حشد من الناس أو الوقوف في صف الانتظار أو في مكان مفتوح أو عند عبور جسر (كوبري) أو في مكان مغلق أو عندما تكون بمفردك خارج أو داخل المنزل أو تسافر في باص, قطار أو سيارة أو تستخدم وسائل النقل العام؟	نعم	لا ←
	**هل تم تسجيل إجابة "نعم" على ٢ أو أكثر من المواقف المذكورة أعلاه في بند E1**	نعم	لا ←

E2	هل تُسبب لك هذه المواقف دائمًا الخوف أو القلق؟	نعم	لا ←
E3	هل تخشى هذه المواقف لدرجة أنك تتجنبها أو تعاني منها أو تحتاج لمن يرافقك لمواجهتها؟	نعم	لا ←
E4	هل هذا الخوف أو القلق مفرط أو غير متناسب مع حجم الخطر الحقيقي في الموقف؟	نعم	لا ←
E5	هل استمر هذا التجنب أو الخوف أو القلق لمدة 6 أشهر على الأقل؟	نعم	لا ←
E6	هل سببت لك هذه الأعراض بمعاناة أو مشاكل ملحوظة في المنزل أو العمل أو اجتماعياً أو في المدرسة أو أثرت عليك بطريقة أخرى ذات أهمية؟	نعم	لا ←
	هل تم تسجيل إجابة "نعم" في البند E6	***نعم***	لا
	نعم= رهاب الأماكن المكشوفة/العامة AGORAPHOBIA		

© 2019 Charité Universitätsmedizin Berlin - Campus Benjamin Franklin. All rights reserved. Translated by Dana Churbaji, Shaymaa Hauary, Sara Mohamed & Carine Karnouk. Supervised by Dr. Malek Bajbouj.

F. اضطراب القلق الاجتماعي (الرهاب الاجتماعي)
SOCIAL PHOBIA (Social Anxiety Disorder)

هذا السهم ← يعني انتقل إلى مربع التشخيص النهائي الخاص بالوحدة واختر (لا) في مربع التشخيص. انتقل بعدها إلى الوحدة التالية.

F1	في الشهر الماضي، هل كان لديك خوف مستمر وقلق ملحوظ من أن تكون محط أنظار أو اهتمام من الآخرين، أو تخشى التعرض للإهانة أو الاحراج أو الرفض؟ يتضمن ذلك أشياء مثل التحدث في الأماكن العامة أو تناول الطعام في الأماكن العامة أو مع الآخرين أو الكتابة بوجود شخص يشاهدك أو الأداء أمام الآخرين أو التواجد في مواقف اجتماعية.	نعم	لا ←
	أمثلة لمواقف اجتماعية تضمن عادة:<br>- البدء أو الاستمرار في حوار<br>- التفاعل والمشاركة في مجموعات صغيرة<br>- التعارف (اللقاءات العاطفية)<br>- التحدث مع أشخاص يشغلون مناصب مسؤولة<br>- حضور الحفلات<br>- التحدث أمام الجمهور<br>- تناول الطعام أمام الآخرين<br>- التبول في الحمامات العامة, ...		
**F2**	هل تُسبب هذه المواقف الاجتماعية دائمًا الخوف أو القلق؟	نعم	لا ←
**F3**	هل تخشى هذه المواقف الاجتماعية لدرجة أنك تتجنبها أو تعاني منها أو تحتاج إلى من يُرافقك لمواجهتها؟	نعم	لا ←
**F4**	هل هذا الخوف أو القلق الاجتماعي مفرط أو غير مُبرر في هذه المواقف الاجتماعية؟	نعم	لا ←
**F5**	هل استمر هذا التجنب الاجتماعي أو الخوف أو القلق لمدة 6 أشهر على الأقل؟	نعم	لا ←
**F6**	هل تسببت هذه المخاوف الاجتماعية في معاناة ملحوظة أو عرقلت قدرتك على الأداء في العمل، أو في المدرسة أو في حياتك الاجتماعية أو في علاقاتك بالآخرين أو أثرت عليك بطريقة أخرى ذات أهمية؟	نعم	لا ←
	هل تم تسجيل إجابة "نعم" عند البند F6 ؟<br>هل تم تسجيل إجابة "نعم" على البند الخاص بـ "استبعاد السبب العضوي" التابع للملخص O2؟	***نعم***	لا
	نعم= اضطراب القلق الاجتماعي (الرهاب الاجتماعي)<br>***SOCIAL PHOBIA (Social Anxiety Disorder)***		
	**مقتصر على الأداء فقط**		☐<br>**SAD فقط**

**ملاحظة للقائم بإجراء المقابلة: يرجى تحديد ما إذا كانت هذه المخاوف مقتصرة على التحدث أو الأداء في الأماكن العامة.**	
اضطراب قلق اجتماعي محصور على الأداء في الأماكن العامة	☐

© 2019 Charité Universitätsmedizin Berlin - Campus Benjamin Franklin. All rights reserved. Translated by Dana Churbaji, Shaymaa Hauary, Sara Mohamed & Carine Karnouk. Supervised by Dr. Malek Bajbouj.

G. اضطراب الوسواس القهري
OBSESSIVE-COMPULSIVE DISORDER

هذا السهم ← يعني انتقل إلى مربع التشخيص النهائي الخاص بالوحدة واختر (لا) في مربع التشخيص. انتقل بعدها إلى الوحدة التالية.

لا ← to G3.a	نعم	في الشهر الماضي، هل كنت منزعجًا من أفكار متكررة أو رغبات مُلحة أو صور غير مرغوب فيها, مقيتة أو مُقلقة أو غير لائقة أو قهرية أو مؤلمة؟ (على سبيل المثال، فكرة أنك متسخ أو ملوث أو أن لديك جراثيم أو الخوف من تلويث الآخرين ، أو الخوف من إ يذاء شخص ما حتي لو كانت الفكرة مُزعجة بالنسبة لك، أو الخوف من تنفيذ بعض الرغبات المُلحة، أو الخوف من أنك ستكون مسؤول عن سير الأمور بشكل خاطئ أو جود وساوس تتعلق بالأفكار الجنسية أو بالصور أو بالرغبات المُلحة أو بوساوس ذات طابع ديني.)	G1. a
لا ← to G3.a	نعم	في الشهر الماضي، هل حاولت قمع هذه الأفكار أو الرغبات المُلحة أو الصور أو تحييدها أو تقليصها ببعض الأفكار أو الأفعال الأخرى؟	G1. b
		**(لا تقُم باحتساب المخاوف المُفرطة بشأن مشاكل الحياة الحقيقية. لا تقُم باحتساب الوساوس المرتبطة مباشرة بالادخار، أو نتف الشعر، أو تقشير الجلد، أو اضطراب تشوه الجسم، أو اضطرابات الأكل، أو الانحرافات الجنسية، أو المقامرة المرضية، أو تعاطي الكحول أو المخدرات لأن المريض قد يستمد المتعة من هذا النشاط وقد ير غب في مقاومته بسبب عواقبه السلبية فقط.)**	
لا	***نعم***	هل تتكرر هذه الهواجس/الوساوس وتعود مجدداً، رغم محاولتك للتخلص منها وتجاهلها؟	G2
		نعم= وساوس<br>***Obsessions***	

لا	***نعم***	في الشهر الماضي ، هل شعرت بدافع لفعل شيء ما بشكل متكرر ردًا على وسواس أو ردًا على قاعدة صارمة ، مثل الغسيل أو التنظيف بشكل مُفرط ، أو عد أو فحص الأشياء مرارًا وتكرارًا ، أو تكرار الأشياء أو ترتيبها ، أو غيرها من الطقوس الغريبة الأخرى ؟	G3. a
لا	***نعم***	هل كان الغرض من القيام بهذه الطقوس منع أو تقليل القلق أو المعاناة أو لمنع حدوث شيء سيء وهل هذه الطقوس مُفرطة أو غير عقلانية؟	G3. b
		نعم= سلوك قهري<br>***Compulsion***	

لا ←	نعم	**هل تم تسجيل إجابة "نعم" على من البنود التالية:**<br>**G3.a + G3.b أو G1.a + G1.b + G2**	

لا	نعم	في الشهر الماضي، هل تسببت هذه الأفكار الوسواسية و / أو السلوكيات القهرية في معاناة ملحوظة، أو أثرت على حياتك في المنزل أو في العمل أو في المدرسة أو في حياتك الاجتماعية أو في علاقاتك بالآخرين أو أثرت عليك بطريقة أخرى ذات أهمية,<br>أو انشغلت بها لأكثر من ساعة في اليوم؟	G4

© 2019 Charité Universitätsmedizin Berlin - Campus Benjamin Franklin. All rights reserved. Translated by Dana Churbaji, Shaymaa Hauary, Sara Mohamed & Carine Karnouk. Supervised by Dr. Malek Bajbouj.

	**وهل تم تسجيل إجابة "نعم" على البند الخاص بـ "استبعاد السبب العضوي" التابع للملخص O2؟**		
	**(تحقق من وجود أي أعراض للوسواس القهري قد بدأت في غضون 3 أسابيع من الإصابة بعدوى)**		
	**حدد مستوى إدراك الشخص للمرض (level of insight) وما إذا كانت النوبة مرتبطة بـمتلازمة لاإرادية "TIC"**		

اضطراب الوسواس القهري - حالي<br>O.C.D Current				
إدراك المرض (level of insight)				
☐<br>جيد أو معقول	☐<br>ضعيف	☐<br>غائب	☐<br>ضلالي	☐<br>ذات صلة بـمتلازمة لاإرادية "TIC"

© 2019 Charité Universitätsmedizin Berlin - Campus Benjamin Franklin. All rights reserved. Translated by Dana Churbaji, Shaymaa Hauary, Sara Mohamed & Carine Karnouk. Supervised by Dr. Malek Bajbouj.

اضطرب كرب ما بعد الصدمة .H
POSTTRAUMATIC STRESS DISORDER

هذا السهم ← يعني انتقل إلى مربع التشخيص النهائي الخاص بالوحدة واختر (لا) في مربع التشخيص. انتقل بعدها إلى الوحدة التالية.

H1	هل سبق لك أن تعرضت أو شهدت أو اضطررت للتعامل مع أحداث صادمة تتضمن الموت الفعلي, خطر الموت أو التهديد بالموت أو إصابة جسدية خطيرة أو التعرض لاعتداء جنسي, بشكل شخصي أو كشاهد على شخص آخر؟<br>تشمل الأمثلة على الأحداث المؤلمة\الصادمة: الحوادث الخطيرة ، الاعتداء الجنسي أو الجسدي ، هجوم إرهابي ، الخطف والأخذ كرهينة، الحريق، الحرب، أو الكوارث الطبيعية، أو مُشاهدة العنف، أو الموت المُفاجئ لشخص قريب منك، أو مرض مهدد للحياة.	نعم	لا ←
H2	بعد الحدث الصادم الذي تعرضت له, هل شعرت بشكل متكرر أنك تعيش الحادثة مجدداً بطريقة مزعجة لا ترغب بها وتسبب لك الضغط والحزن (كمشاهدة أحلام/كوابيس متكررة تخصّ الحادثة, أو استرجاع الذكريات بشدّة أو استرجاع صور من الحادث كما لو أنه يحدث مجدداً)؟ أو هل كان لديك ردود فعل جسدية أو نفسية شديدة عندما يتم تذكيرك بالحدث أو عند تعرضك لحدث مشابه؟	نعم	لا ←
H3	<u>في الشهر الماضي:</u>		
H3. a	هل كنت تحاول بإستمرار تجنب التفكير أو تذكُر التفاصيل أو المشاعر المؤلمة المرتبطة بالحدث؟	نعم	لا
H3. b	هل كنت تحاول بإستمرار تجنب الأشخاص أو المحادثات أو الأماكن أو المواقف أو الأنشطة أو الأشياء التي تُعيد إليك ذكريات مؤلمة عن الحدث؟	نعم	لا
	**هل تم تسجيل "نعم" على ١ أو أكثر من أسئلة البند H3؟**	نعم	لا ←
H4	<u>في الشهر الماضي:</u>		
H4. a	هل واجهتك مشكلة في استرجاع جزء مهم من الحدث الصادم؟<br>(يجب أن لا يتعلق سبب عدم القدرة على الاسترجاع بالآتي: إصابة في الرأس أو تناول الكحول أو المواد المُخدرة/العقاقير)	نعم	لا
H4. b	هل تملكتك بشكل مستمر وغير منطقي, مشاعر سلبية تجاه نفسك أو الآخرين أو العالم؟	نعم	لا
H4. c	هل كنت بطريقة غير منطقية تلوم نفسك أو تلوم الآخرين على ما تعرضت له؟	نعم	لا
H4. d	هل كانت مشاعرك سلبية بشكل دائم؟ (كمشاعر الخوف, الرعب, الغضب, الشعور بالذنب, الخجل/الخزي)	نعم	لا
H4. e	هل أصبحت أقل اهتماماً بشكل ملحوظ بالمشاركة بأنشطة كانت تعني لك الكثير سابقاً؟	نعم	لا
H4. f	هل كنت تشعر بأنك منفصل أو مُنعزل عن الآخرين؟	نعم	لا
H4. g	هل كنت غير قادر على الشعور بأي مشاعر إيجابية (مثل السعادة أو الرضى أو مشاعر الحب)؟	نعم	لا
	**هل تم تسجيل إجابة "نعم" على ٢ أو أكثر من أسئلة البند H4؟**	نعم	لا ←
H5	<u>في الشهر الماضي:</u>		
H5. a	هل كنت سريع الغضب بشكل أكبر أو كانت لديك نوبات غضب ناتجة عن استفزاز بسيط أو بدون سبب على الاطلاق؟	نعم	لا

© 2019 Charité Universitätsmedizin Berlin - Campus Benjamin Franklin. All rights reserved. Translated by Dana Churbaji, Shaymaa Hauary, Sara Mohamed & Carine Karnouk. Supervised by Dr. Malek Bajbouj.

H5. b	هل كنت أكثر تهوراً أو تصرفت بطريقة تضرك أوتؤذيك؟	نعم	لا
H5. c	هل كنت متوتراً, متأهباً (أو على أعصابك) باستمرار؟	نعم	لا
H5. d	هل كنت ترتعب بسهولة (من السهل ترويعك)؟	نعم	لا
H5. e	هل كنت تواجه صعوبات أكبر في التركيز؟	نعم	لا
H5. f	هل كنت تواجه صعوبات أكبر في النوم؟	نعم	لا
	**هل تم تسجيل إجابة "نعم" على ٢ أو أكثر من الأسئلة التابعة للبند H5؟**	نعم	لا ←
H6	هل بدأت كل هذه المشاكل بعد تعرضك للحدث الصادم واستمرت لأكثر من شهر؟	نعم	لا ←
H7	خلال الشهر الماضي ، هل تسببت هذه المشكلات في معاناه ملحوظة أو عرقلت حياتك في المنزل أو في العمل أو في المدرسة أو في حياتك الاجتماعية أو في علاقاتك بالآخرين أو أثرت عليك بطريقة أخرى ذات أهمية؟	نعم	لا
	وهل تم تسجيل إجابة "نعم" على البند الخاص بـ "استبعاد السبب العضوي" التابع للملخص O2؟		
	حدد ما إذا كانت الحالة مرتبطة بالانفصال عن الذات أو عدم الوعي بالذات (depersonalization) أو الانفصال عن الواقع أو التغيير في إدراك الواقع (Derealization) أو تأخر في التعبير (Delayed Expression).		

اضطراب كرب ما بعد الصدمة – حالي<br>POSTTRAUMATIC STRESS DISORDER<br>CURRENT		
مع		
☐<br>انفصال عن الذات أو عدم الوعي بالذات<br>(depersonalization)	☐<br>انفصال عن الواقع أو تغُير في إدراك الواقع<br>(Derealization)	☐<br>تأخر في التعبير<br>(Delayed Expression)

© 2019 Charité Universitätsmedizin Berlin - Campus Benjamin Franklin. All rights reserved. Translated by Dana Churbaji, Shaymaa Hauary, Sara Mohamed & Carine Karnouk. Supervised by Dr. Malek Bajbouj.

اضطراب تعاطي الكحول .I
ALCOHOL USE DISORDER

هذا السهم ← يعني انتقل إلى مربع التشخيص النهائي الخاص بالوحدة واختر (لا) في مربع التشخيص.
انتقل بعدها إلى الوحدة التالية.

I1	خلال الأشهر الـ ١٢ الماضية، هل شربت ثلاثة أنواع من المشروبات الكحولية أو أكثر, خلال ٣ ساعات، وحدث ذلك في ثلاث مناسبات أو أكثر؟	نعم	لا ←
I2	خلال الأشهر الـ ١٢ الماضية		
a.	خلال الأوقات التي كنت تشرب فيها الكحول ، هل كان ينتهي بك الأمر إلى شرب كمية أكبر من التي كنت تنوي أو تُخطط لتناولها عندما بدأت بالشرب؟	نعم	لا
b.	هل أردت مرارا التقليل أو السيطرة على شرب الكحول؟<br>هل حاولت التقليل من أو السيطرة على شرب الكحول، لكنك لم تستطع؟	نعم	لا
c.	الأيام التي كنت تشرب فيها الكحول، هل استغرقت وقتا طويلاً في الحصول على الكحول، شرب الكحول, أو التعافي من تأثير الكحول؟	نعم	لا
d.	هل كنت تشتهي شرب الكحول أو كانت لديك رغبة قوية أو مُلحة في شرب الكحول؟	نعم	لا
e.	هل قضيت وقتاً أقل في القيام بمسؤولياتك في العمل أو المدرسة أو المدرسة بسبب شربك المتكرر للكحول؟	نعم	لا
f.	إن كان شربك للكحول قد سبب لك مشاكل مع أسرتك أو مع أشخاص آخرين, فهل ما زلت مستمراً في شرب الكحول رغم ذلك؟	نعم	لا
g.	هل كنت في حالة سكر في أي موقف كنت ستتعرض فيه أنت أو غيرك لخطر جسدي، أكثر من مرة؟(على سبيل المثال ، قيادة سيارة ، أو ركوب دراجة نارية ، أو استخدام الآلات ، أو ركوب القوارب ، وما إلى ذلك)	نعم	لا
h.	هل استمريت في شرب الكحول, على الرغم من أنه كان من الواضح أنه فاقم من أو تسبب في مشكلات نفسية أو جسدية؟	نعم	لا
i.	هل قللت أو تخليت عن القيام بعمل هام أو أنشطة اجتماعية أو ترفيهية بسبب شربك؟	نعم	لا
j.	هل كنت بحاجة الى شرب كمية أكبر لتحصل على نفس التأثير الذي حصلت عليه اول مرة عندما بدأت بشرب الكحول او هل حصلت على تأثير اقل بكثير مقارنة بالتأثير الذي حصلت عليه عند شربك لأول مرة لنفس الكمية	نعم	لا
k1	عندما خفضت من الإفراط في شرب الكحول بكمية كبيرة أو لفترة طويلة, هل كانت لديك أي من الأعراض التالية:<br>**إذا تم تسجيل إجابة "نعم" على ٢ أو أكثر من الأعراض الـ ٨ المذكورة أعلاه, قم بتسجيل "نعم" على البند K1**	نعم	لا
	1. زيادة التعرق أو زيادة معدل ضربات القلب	☐	
	2. رعشة في اليد (رجفة)	☐	
	3. صعوبة في النوم	☐	
	4. الغثيان أو القيء	☐	
	5. سماع أو رؤية أشياء لا يُمكن أن يراها أو يسمعها الآخرون أو وجود أحاسيس في الجلد دون سبب واضح أو مثير واضح	☐	
	6. الانفعال	☐	

© 2019 Charité Universitätsmedizin Berlin - Campus Benjamin Franklin. All rights reserved. Translated by Dana Churbaji, Shaymaa Hauary, Sara Mohamed & Carine Karnouk. Supervised by Dr. Malek Bajbouj.

	7. القلق والتوتر	☐		
	8. اختلاجات (تشنجات)	☐		
k2	هل كنت تشرب الكحول للتخفيف من أو لتجنب أعراض الانسحاب أو لتفادي الـ Hung-over(حالة بدنية مؤقتة ومزعجة، تتميز عادة بالصداع الشديد والغثيان)؟		نعم	لا
I2.k<br>Summary	**إذا تم تسجيل إجابة "نعم" على I2.k1 أو I2.k2, اختر الإجابة نعم**		نعم	لا

	**هل تم تسجيل "نعم" على إجابتين أو أكثر من أسئلة البند I2**<br>**(من I2.a حتى SUMMARY-I2.k)**<br>**(يتم احتساب كلاً من البند i2k1 و البند i2k2 معًا كـ بند واحد من بين هذه الخيارات)**	***نعم***	لا

اضطراب تعاطي الكحول - في الأشهر الـ١٢ الماضية

ALCOHOL USE DISORDER - PAST 12 MONTHS

محددات شدة اضطراب تعاطي الكحول	
خفيف = 2-3 أعراض من البندI2	
متوسطة = 4-5 أعراض من البند I2	
شديد = 6 أو أكثر من أعراض البند I2	
في مرحلة تعافي مبكر = لم يتم اكتمال معايير التشخيص لمدة تتراوح بين 3 و 12 شهرًا.	يستثنى من مرحلتين التعافي المعيار "د" أعلاه [اللهفة أو الاشتهاء أو الاشتياق (craving)]
في مرحلة تعافي مستمر = لم يتم اكتمال معايير التشخيص لمدة 12 شهرا أو أكثر.	
في بيئة خاضعة للرقابة = حيث يتم تقييد الوصول إلى الكحول.	

حدد شدة الاضطراب					
خفيف<br>☐	متوسط<br>☐	شديد<br>☐	في مرحلة تعافي مبكر<br>☐	في مرحلة تعافي مستمر<br>☐	في بيئة خاضعة للرقابة<br>☐

© 2019 Charité Universitätsmedizin Berlin - Campus Benjamin Franklin. All rights reserved. Translated by Dana Churbaji, Shaymaa Hauary, Sara Mohamed & Carine Karnouk. Supervised by Dr. Malek Bajbouj.

J. اضطراب استخدام المواد المخدرة (غير الكحولية)
SUBSTANCE USE DISORDER (NON-ALCOHOL)

هذا السهم ← يعني انتقل إلى مربع التشخيص النهائي الخاص بالوحدة واختر (لا) في مربع التشخيص. انتقل بعدها إلى الوحدة التالية.

		سأعرض عليك الآن قائمة من العقاقير أو المواد المُخدرة التي تُباع في الشارع:	
لا ←	نعم	خلال الأشهر الـ ١٢ الماضية، هل تناولت أيًا من هذه الأدوية/المواد المُخدرة أكثر من مرة ، لكي تشعر بالانتشاء، أو للشعور بالابتهاج، أو للحصول على مزاج عالي جداً أو لتُغير من حالتك المزاجية؟	J1

ضع دائرة حول كل عقار/مخدر يتم تعاطيه:

Stimulants: amphetamines, "speed", crystal meth, “crank”, Dexedrine, Ritalin, diet pills.	المنشطات: الأمفيتامينات ، "سبيد" ، الكريستال ميث ، "كرنك" ، "راش" ، ديكسيدرين ، ريتالين ، حبوب للحمية الغذائية.
Cocaine: snorting, IV, freebase, crack, "speedball".	الكوكايين: عن طريق الاستنشاق، الحقن في الوريد، فريباس (الدخان الناتج من حرق بودرة الكوكايين)، كراك (بلورات الكوكايين)، "سبيد بول" تعاطي الكوكايين مع المورفين أو الهرويين في حقنة واحدة.
Opiates: heroin, morphine, Dilaudid, opium, Demerol, methadone, Darvon, codeine, Percodan, Vicodin, OxyContin.	المواد الأفيونية: الهيروين ، المورفين ، الدايلوديد ، الأفيون (الخشخاش)، الديرمول ، الميثادون ، الدارفون ، الكوديين ، البيركودان ، الفيكودين ، الأوكسي كونتين.
Hallucinogens: LSD ("acid"), mescaline, peyote, psilocybin, STP, "mushrooms", “ecstasy”, MDA, MDMA.	المهلوسات: (حمض) الـLSD ، و الميسكالين، والبيوت، و بيسايلوسيبين و الـSTP و " فطرعش الغراب" و "الإكستاسي (حبة السعادة)" و الـ MDA والـ MDMA.
Dissociative Drugs: PCP (Phencyclidine ,"Angel Dust", "Peace Pill", “Hog”), or ketamine (“Special K”).	العقاقير الإنفصالية: PCP (فينسيكليدين، "غبار الملاك"، "حبة السلام"، "الخنزير")، أو الكيتامين (Special K).
Inhalants: "glue", ethyl chloride, “rush”, nitrous oxide ("laughing gas"), amyl or butyl nitrate ("poppers").	المستنشقات (المواد الطيارة): "الغراء/الكُله/الصمغ" ، كلوريد الإيثيل ، "الراش" ، أكسيد النايتروز ("غاز الضحك") ، الأميل أو نترات البوتيل ("بوبيرس").
Cannabis: marijuana, hashish ("hash"), THC, "pot", "grass", "weed", "reefer".	القِنَب الهندي: المايجوانا، الحشيش ("الهاش") ،الـ THC ، "وعاء القنب"، "عشبة القنب" "الكيف"، "ساق القنب" (البانجو)، "سيجارة من القنب الهندي".
Sedatives, Hypnotics or Anxiolytics: Quaalude, Seconal ("reds"), Valium, Xanax, Librium, Ativan, Dalmane, Halcion, barbiturates, Miltown, GHB, Roofinol, “Roofies”.	المهدئات ، المنومات أو مضادات القلق: كوالود، سيكونال، فاليوم، زاناكس، ليبريوم، أتيفان، دالمان، هالسيون، الباربيتيوراتس، ميل تاون، الـ GHB، روفينول، "روفييس أو روهيبنول".
Miscellaneous: steroids,tramadol, nonprescription sleep or diet pills. Cough Medicine? Any others?	أدوية اخرى متنوعة: ستيرويدز، ترامادول، حبوب للحمية الغذائية أو النوم غير موصوفة من قبل طبيب. دواء السعال "الكحة" أو أي أدوية آخري؟

حدد الأدوية/ المخدرات الأكثر استخداماً:
ما هو المخدر (المخدرات) الذي يُسبب أكبر المشاكل؟

© 2019 Charité Universitätsmedizin Berlin - Campus Benjamin Franklin. All rights reserved. Translated by Dana Churbaji, Shaymaa Hauary, Sara Mohamed & Carine Karnouk. Supervised by Dr. Malek Bajbouj.

**ابدأ بالاستفسار عن المعايير الموضحة أدناه للعقار/المخدر الذي يسبب أكبر المشاكل والأكثر احتمالاً أن يفي بمعايير التشخيص**
**لتشخيص اضطراب تعاطي المخدرات/العقاقير, وفي حال تعاطي عدة فئات من المخدرات/العقاقير: استكشف أكبر عدد ممكن أو أصغر عدد ممكن من الفئات, وذلك وفقاً للبروتوكول الذي تعمل على أساسه.**

J2	نظراً لتعاطيك (اسم الدواء أو المخدر/ صنف الدواء أو المخدر), في الأشهر الـ ١٢ الماضية:	نعم	لا ←
a.	خلال الأوقات التي كنت تتعاطى فيها لـ (اسم الدواء أو المخدر/ صنف الدواء أو المخدر),هل كان ينتهي بك الأمر إلى شرب كمية أكبر من التي كنت تنوي أو تخطط لتعاطيها عندما بدأت؟	نعم	لا
b.	هل حاولت بشكل متكرر التقليل أو السيطرة على تعاطيك لـ (اسم الدواء أو المخدر/ صنف الدواء أو المخدر)؟<br>هل حاولت أن تقلل او تسيطر على تعاطيك لـ (اسم الدواء أو المخدر/ صنف الدواء أو المخدر) لكنك لم تستطع؟<br>**إذا كانت الإجابة بنعم على أيهما ، فاكتب نعم.**	نعم	لا
c.	في الأيام التي كنت تتعاطى فيها من (اسم الدواء أو المخدر/ صنف الدواء أو المخدر), هل استغرقت وقتا طويلاً في الحصول على الدواء/ المخدر, تناوله أو التعافي من تأثيره؟	نعم	لا
d.	هل كنت تشتهي أو كانت لديك رغبة قوية أو مُلحة في تعاطي (اسم الدواء أو المخدر/ صنف الدواء أو المخدر)؟	نعم	لا
e.	هل قضيت وقتاً أقل في القيام بواجباتك في العمل أو المدرسة أو في المنزل بسبب تعاطيك المتكرر لـ (اسم الدواء أو المخدر/ صنف الدواء أو المخدر)؟	نعم	لا
f.	إذا كان تعاطيك لـ (اسم الدواء أو المخدر/ صنف الدواء أو المخدر) قد تسبب لك بمشاكل مع أسرتك أو مع أشخاص آخرين، هل ما زلت مستمرا في تعاطيه رغم تلك المشاكل؟	نعم	لا
g.	هل تعاطيت (اسم الدواء أو المخدر/ صنف الدواء أو المخدر) في مواقف كنت ستتعرض فيها أنت أو غيرك لخطر جسدي, أكثر من مرة؟ (قيادة سيارة ، أو ركوب دراجة نارية ، أو استخدام الآلات ، أو ركوب القوارب ، وما إلى ذلك)	نعم	لا
h.	هل استمريت في تعاطي (اسم الدواء أو المخدر/ صنف الدواء أو المخدر) على الرغم من أنه كان من الواضح أنه فاقم من أو تسبب في مشكلات نفسية أو جسدية؟	نعم	لا
i.	هل قللت أو تخليت عن القيام بعمل هام أو أنشطة اجتماعية أو ترفيهية بسبب تعاطيك لـ (اسم الدواء أو المخدر/ صنف الدواء أو المخدر)؟	نعم	لا
j.	هل كنت بحاجة إلى تعاطي المزيد من (أسم الدواء أو المادة المُخدرة / صنف المُخدر أو الدواء) من أجل الحصول على نفس التأثير الذي حصلت عليه عندما بدأت في تعاطيه لأول مرة أو هل بدأت في الحصول على تأثير أقل بكثير من الذي حصلت عليه أول مرة مع الاستمرار في استخدام نفس الكمية؟<br>**يتم تسجيل الإجابة على هذا المعيار بـ "لا" إذا تم وصف الدواء واستخدامه تحت إشراف طبي مناسب.**	نعم	لا

k1	عندما خفضت من الإفراط في التعاطي بكمية كبيرة أو لفترة طويلة, هل تعرضت لأحد الأعراض لانسحابيه التالية:<br>**إذا كانت الإجابة بنعم على العدد المطلوب من أعراض الانسحاب للفئة المحددة، فقم بتسجيل "نعم" عند البند j2k1 .**<br>**يتم تسجيل الإجابة على هذا المعيار بـ لا إذا تم وصف الدواء واستخدامه تحت اشراف طبي مناسب.**	نعم	لا

المهدئات، المنومات أو مضادات القلق (2 أو أكثر من أعراض الانسحاب التالية)	
1. زيادة التعرق أو زيادة معدل ضربات القلب ،	☐
2. رعشة في اليد (رجفة)	☐
3. صعوبة في النوم	☐
4. الإسهال والإقياء	☐

© 2019 Charité Universitätsmedizin Berlin - Campus Benjamin Franklin. All rights reserved. Translated by Dana Churbaji, Shaymaa Hauary, Sara Mohamed & Carine Karnouk. Supervised by Dr. Malek Bajbouj.

5. سماع أو رؤية أشياء لا يُمكن أن يراها أو يسمعها الآخرون أو وجود أحاسيس في الجلد دون سبب أو مُثير واضح.	☐
6. الانفعال	☐
7. القلق والتوتر	☐
8. اختلاجات (تشنجات)	☐

الأفيونيات (3 أو أكثر من أعراض الانسحاب التالية)	
1. الشعور بالاكتئاب	☐
2. الغثيان أو القيء	☐
3. آلام العضلات	☐
4. سيلان الأنف أو العيون الدامعة	☐
5. اتساع حدقة العين، أو نتوءات الجلد (مُشابه لجلد الوزة) أو وقوف شعر الجسم أو التعرق	☐
6. الإسهال	☐
7. التثاؤب	☐
8. الهبات الساخنة (شعور مُفاجئ بالحرارة أو السخونة)	☐
9. صعوبة في النوم	☐

المنشطات والكوكايين (2 أو أكثر من أعراض الانسحاب التالية)	
1. التعب والإجهاد	☐
2. أحلام يقظة أو أحلام مزعجة	☐
3. صعوبة في النوم أو الافراط في النوم	☐
4. زيادة الشهية	☐
5.الظهور أوالشعور بالبطء عقليا وجسديا	☐

القنب (3 أو أكثر من أعراض الانسحاب التالية)	
1. سرعة الانفعال \ التهيُج أو الغضب أو العدوانية	☐
2. العصبية أو القلق	☐
3.اضطرابات النوم	☐
4. فقدان الشهية أو فقدان الوزن	☐
5. التململ (عدم الشعور بالراحة في أي وضع - "ضايج")	☐
6. الشعور بالاكتئاب	☐
7. انزعاج كبير من أحد الأعراض التالية: "ألم في المعدة" ، الرعشة أو "الرجفة" ، التعرق ، الهبات .الساخنة ، القشعريرة ، الصداع.	☐

J2. k2	هل كنت تتعاطى(اسم الدواء أو المخدر/ صنف الدواء أو المخدر) للتخفيف من أو لتجنب أعراض الانسحاب؟	نعم	لا
J2.k Summary	**إذا تم تسجيل إجابة "نعم" على J2.k1 أو J2.k2, اختر إجابة نعم**	نعم	لا

	**هل تم تسجيل إجابة "نعم" على إجابتين أو أكثر من أسئلة البند J2 (منJ2.a حتى J2.k-SUMMARY) (يتم احتساب كلاً من البند j2k1 و البند j2k2 معًا كـ بند واحد من بين هذه الخيارات)**	***نعم***	لا
في الأشهر الـ١٢ الماضية – اضطراب تعاطي المواد المخدرة (اسم الدواء أو المخدر/ صنف الدواء أو المخدر)<br>SUBSTANCE<br>(Drug or Drug Class Name)<br>USE DISORDER - PAST 12 MONTHS			

محددات شدة اضطراب تعاطي المخدرات:
خفيف = 2-3 أعراض من البندJ2
متوسطة = 4-5 أعراض من البند J2
شديد = 6 أو أكثر من أعراض البند J2

© 2019 Charité Universitätsmedizin Berlin - Campus Benjamin Franklin. All rights reserved. Translated by Dana Churbaji, Shaymaa Hauary, Sara Mohamed & Carine Karnouk. Supervised by Dr. Malek Bajbouj.

في مرحلة تعافي مبكر = لم يتم اكتمال معايير التشخيص لمدة تتراوح بين 3 و 12 شهرًا.	يستثنى من مرحلتين التعافي المعيار "د" أعلاه [اللهفة أو الاشتهاء أو الاشتياق (craving) للمخدر/العقار]
في مرحلة تعافي مستمر = لم يتم اكتمال معايير التشخيص لمدة 12 شهرا أو أكثر.	
في بيئة خاضعة للرقابة = حيث يتم تقييد الوصول إلى الكحول.	

حدد شدة الاضطراب					
خفيف ☐	متوسط ☐	شديد ☐	في مرحلة تعافي مبكر ☐	في مرحلة تعافي مستمر ☐	في بيئة خاضعة للرقابة ☐

© 2019 Charité Universitätsmedizin Berlin - Campus Benjamin Franklin. All rights reserved. Translated by Dana Churbaji, Shaymaa Hauary, Sara Mohamed & Carine Karnouk. Supervised by Dr. Malek Bajbouj.

الاضطرابات الذهانية واضطرابات المزاج مع ملامح ذهانية .K
PSYCHOTIC DISORDERS AND MOOD DISORDER WITH PSYCHOTIC FEATURES

هذا السهم ← يعني انتقل إلى مربع التشخيص النهائي الخاص بالوحدة واختر (لا) في مربع التشخيص. انتقل بعدها إلى الوحدة التالية.

**اسأل عن مثال على كل سؤال تمت الإجابة عليه بشكل إيجابي. سجل "نعم" فقط إذا كانت الأمثلة تُظهر بوضوح تدهور واضطراب التفكير أو الإدراك أو إذا لم تكن مناسبة (اجتماعياً) ثقافياً. الغرض من هذه الوحدة هو استبعاد المرضى الذين يعانون من اضطرابات ذهانية. هذه الوحدة تحتاج إلى خبرة.**	

	سأطرح عليك الآن عدة أسئلة عن بعض التجارب الغير معتادة التي يعيشها أو يمر بها بعض الأشخاص.		
K1.a	هل اعتقدت يوماً أن الناس يتجسسون عليك أو أن شخصاً ما يدبر لك مكيدة أو يحاول إلحاق الضرر بك؟<br>**ملاحظة: اطلب أمثلة لاستبعاد المطاردة/التجسس/الملاحقة الفعلية/الواقعية في حياة الشخص.**	نعم	لا
K1.b	إذا كانت الإجابة "نعم": هل ما زلت تعتقد ذلك في الوقت الحالي؟	نعم	لا
K2.a	هل اعتقدت يوماً ما أن شخصا ما يستطيع قراءة أو سماع أفكارك، أو أن باستطاعتك قراءة أو سماع أفكار شخص آخر؟	نعم	لا
K2.b	**إذا كانت الإجابة "نعم":** هل ما زلت تعتقد ذلك في الوقت الحالي؟	نعم	لا
K3.a	هل اعتقدت يوماً أن شخصاً ما أو قوة خارجية ما قد وضعت أفكاراً في عقلك, أنت لست مصدرها، أو جعلتك تتصرف بطريقة مختلفة عن المعتاد؟ هل شعرت يوماً بأنك مسكون (من الجن مثلا)؟<br>**القائم بإجراء المقابلة:** اسأل عن أمثلة واحذف الأمثلة التي لا تعتبر ذهانية	نعم	لا
K3.b	إذا كانت الإجابة "نعم": هل ما زلت تعتقد ذلك في الوقت الحالي؟	نعم	لا
K4.a	هل اعتقدت يوماً بوجود رسائل خاصة يتم إرسالها لك من خلال التلفزيون، الراديو، الإنترنت، الصحف, الكتب أو المجلات، أو أن هناك شخصاً لا تعرفه شخصياً مهتم بك بشكل خاص؟	نعم	لا
K4.b	إذا كانت الإجابة "نعم": هل ما زلت تعتقد ذلك في الوقت الحالي؟	نعم	لا
K5.a	هل اعتقد أقاربك أو أصدقائك يوماً أن معتقداتك غريبة أو غير عادية؟<br>**القائم بإجراء المقابلة: اسأل عن أمثلة.**<br>**سجل "نعم" فقط إذا كانت الأمثلة من الأفكار الضلالية بشكل واضح ولم يتم استكشافها في أسئلة البنود K1 إلى K4. على سبيل المثال، الضلالات الدينية أو الضلالات المتعلقة بالوفاة أو الضلالات المتعلقة بالمرض أو الضلالات الجسدية أو ضلالات العظمة أو الغيرة أو الذنب أو الفشل أو عدم الكفاءة أو الخراب أو الفقر أو الضلالات العدمية.**	نعم	لا
K5.b	**إذا كانت الإجابة "نعم":** هل مازالوا يعتبرون أن معتقداتك غريبة أو غير عادية في الوقت الحالي؟	نعم	لا
K6.a	هل سمعت يوماً أشياء لم يستطع الآخرون سماعها، كالأصوات مثلاً؟	نعم	لا
	إذا كانت الإجابة على الهلوسة السمعية "نعم", اسأل، هل سمعت الصوت يُعلق على أفكارك أو سلوكك أو هل سمعت صوتين أو أكثر يتحدثون مع بعضهم البعض؟	نعم	لا
K6.b	إذا كانت الإجابة "نعم" على البند K6.a, اسأل:<br>هل سمعت هذه الأصوات في الشهر الماضي؟	نعم	لا
	إذا كانت الإجابة على الهلوسة السمعية "نعم", اسأل، هل سمعت الصوت يُعلق على أفكارك أو سلوكك أو هل سمعت صوتين أو أكثر يتحدثون مع بعضهم البعض؟	نعم	لا
K7.a	هل شاهدت يوماً رؤية وأنت مستيقظ أو شاهدت أشياء لا يمكن للآخرين رؤيتها؟<br>للقائم **بإجراء المقابلة:** تحقق ما إذا كانت الهلوسة البصرية غير مناسبة اجتماعياً (ثقافياً)	نعم	لا
K7.b	إذا كانت الإجابة "نعم", هل رأيت هذه الأشياء في الشهر الماضي؟	نعم	لا

© 2019 Charité Universitätsmedizin Berlin - Campus Benjamin Franklin. All rights reserved. Translated by Dana Churbaji, Shaymaa Hauary, Sara Mohamed & Carine Karnouk. Supervised by Dr. Malek Bajbouj.

	التقييم بناء على رأي القائم بإجراء المقابلة:		
K8.a	**هل سبق للمريض في الماضي أن كان حديثة أو كلامه غير منتظم أو غير متناسق أو يخرج عن المسار، أو هل كان ملحوظًا فيه عدم تسلسل الأحداث وفقدان الروابط في الحديث؟**	نعم	لا
K8.b	**هل يظهر المريض حالياً (أثناء المقابلة) حديث أو كلام غير منتظم أو غير متناسق أو يخرج عن المسار، أو هل يلاحظ في حديثه عدم تسلسل الأحداث وفقدان الروابط في الحديث؟**	نعم	لا
K9.a	**هل أبدى المريض في الماضي سلوكًا غير منتظم أو تخشبي (كتاتوني)؟**	نعم	لا
K9.b	**هل يُظهر المريض حاليًا (أثناء المقابلة) سلوكاً غير منتظم أو تخشبي (كتاتوني)؟**	نعم	لا
K10.a	**هل كان لدى المريض في الماضي أعراض سلبية، على سبيل المثال انخفاض كبير في التعبير العاطفي/الانفعالي أو السطحية العاطفية/الانفعالية، ضعف شديد أو الافتقار إلى الكلام (ALOGIA) أو عدم القدرة على البدء أو الاستمرار في أنشطة موجهة نحو هدف معين (AVOLITION)؟**	نعم	لا
K10.b	**هل يظهر المريض خلال المقابلة أعراض سلبية للفصام، على سبيل المثال انخفاض كبير في التعبير العاطفي/الانفعالي أو السطحية العاطفية/الانفعالية، ضعف شديد أو الافتقار إلى الكلام (ALOGIA) أو عدم القدرة على البدء أو الاستمرار في أنشطة موجهة نحو هدف معين (AVOLITION)؟**	نعم	لا

K11.a	**هل تم تسجيل إجابة "نعم" على سؤال واحد أو أكثر من الأسئلة a في البنود K1.a حتى البند K7.a**		نعم	لا<br>←<br>K13
	**هل تم تسجيل إجابة "نعم" في مربع تشخيص أي من هذه الاضطرابات؟**			
	**نوبة اكتئابية كبرى (حالية، متكررة أو ماضية)**	أو نوبة هوس أو نوبة هوس خفيف (حالية أو ماضية)		
	**كم من الوقت استمرت نوبة المزاج؟**			
	**كم من الوقت استمرت نوبة الذهان؟**			
	**في حال وجود نوبة مزاجية، لا تقم بتسجيل إجابة "نعم" على البند K11.a إلا ف حالة وجود اضطراب مزاج في أغلب الوقت من المدة الإجمالية للأعراض الذهانية. خلاف ذلك, سجل "لا".**			
	**إذا كانت الإجابة "لا" على البند K11.a وكانت المدة الإجمالية لنوبة المزاج أقل من المدة الإجمالية للنوبة الذهانية، فاختر "لا" في كل من مربعات التشخيص الخاصة بـ "اضطراب المزاج مع ملامح ذهانية وانتقل إلى البند K13**			

K11.b	أخبرتني في وقت سابق, أنك مررت بفترة (فترات) شعرت فيها بالاكتئاب/ النشوة والابتهاج /الانفعال بشكل مستمر	نعم	لا
	هل المعتقدات والتجارب التي ذكرتها للتو (الأعراض المذكورة في البنودL7.a حتى K1.a) تقتصر على الفترات التي شعرت فيها بالاكتئاب/ النشوة والابتهاج /الانفعال بشكل مستمر؟		
	**إذا كان المريض قد مر بفترة لا تقل عن أسبوعين مع وجود المعتقدات والتجارب (الأعراض الذهانية) المذكورة لمدة لا تقل عن أسبوعين دون وجود اكتئاب/ نشوة وابتهاج /الانفعال بشكل مستمر, وقتها, اختر الإجابة "لا" لهذا الاضطراب.**		
	**إذا كانت الإجابة "لا" على مجموعة هذه الاضطرابات, فاختر "لا" للبند K12 وانتقل إلى البند K13**		
	اضطراب المزاج مع ملامح ذهانية – مزمن مدى الحياة<br>MOOD DISORDER WITH PSYCHOTIC FEATURES LIFETIME		

K12.a	**هل تم تسجيل "نعم" على سؤال واحدة أو أكثر من الأسئلة b في البنود K1.b حتى البند K7.b**<br>**وهل تم تسجيل إجابة "نعم" في مربع تشخيص أي من هذه الاضطرابات؟**		نعم	لا
	**هل تم تسجيل "نعم" في مربع تشخيص أي من هذه الاضطرابات؟**			
	**نوبة اكتئابية كبرى (حالية)**	أو نوبة هوس أو نوبة هوس خفيف (حالية)		
	**إذا تم تسجيل إجابة "نعم" في مربع تشخيص الاضطراب المزاجي مع ملامح ذهانية (حالي أو مدى الحياة), اختر لا للبنود K13 و K14 وانتقل إلى الوحدة التالية.**			

© 2019 Charité Universitätsmedizin Berlin - Campus Benjamin Franklin. All rights reserved. Translated by Dana Churbaji, Shaymaa Hauary, Sara Mohamed & Carine Karnouk. Supervised by Dr. Malek Bajbouj.

اضطراب المزاج مع ملامح ذهانية – حالي<br>MOOD DISORDER WITH<br>PSYCHOTIC FEATURES<br>CURRENT

K13	**هل تم تسجيل "نعم" على سؤال واحدة أو أكثر من الأسئلة b في البنود K1.b حتى البند K8.b**<br>**وهل تم تسجيل "نعم" في مربع تشخيص أي من هذه الاضطرابات؟**<br>**و هل تم تسجيل "نعم" على ٢ أو أكثر من الأسئلة b من البنود K1.b حتى البندK10.b؟**<br>**وهل ظهرت ٢ على الأقل من الأعراض الذهانية خلال نفس الشهر؟**<br>**هل تم تسجيل إجابة "نعم" على البند الخاص بـ "استبعاد السبب العضوي" التابع للملخص O2؟**	نعم	لا
اضطراب ذهاني - حالي<br>MOOD DISORDER WITH<br>PSYCHOTIC FEATURES<br>CURRENT			

K14	**هل تم تسجيل إجابة "نعم" في البند K13؟**<br>**و هل تم تسجيل إجابة "نعم" لواحدة أو أكثر من الأسئلة a في البنود K1.a حتى K8.a؟**<br>**و هل تم تسجيل إجابة "نعم" لـ ٢ أو أكثر من الأسئلة a في البنود K1.a حتى K10.a؟**<br>**و هل ظهرت ٢ على الأقل من الأعراض الذهانية خلال نفس الشهر؟**<br>**و هل تم تسجيل إجابة "نعم" على البند الخاص بـ "استبعاد السبب العضوي" التابع للملخص O2؟**	نعم	لا
اضطراب ذهاني – مزمن مدى الحياة<br>MOOD DISORDER WITH<br>PSYCHOTIC FEATURES<br>LIFETIME			

© 2019 Charité Universitätsmedizin Berlin - Campus Benjamin Franklin. All rights reserved. Translated by Dana Churbaji, Shaymaa Hauary, Sara Mohamed & Carine Karnouk. Supervised by Dr. Malek Bajbouj.

فقدان الشهية العصبي .L
ANOREXIA NERVOSA

هذا السهم ← يعني انتقل إلى مربع التشخيص النهائي الخاص بالوحدة واختر (لا) في مربع التشخيص. انتقل بعدها إلى الوحدة التالية.

L1. A	ما هو طولك؟		
L1. B	ما هو أقل وزن كنت عليه في الأشهر الثلاثة الماضية؟		
L1. C	**هل وزن المريض مساوي لـ أو يقل عن الحد الأدنى المقابل لطوله / طولها؟ (انظر الجدول أدناه)**	نعم	لا ←

	خلال الأشهر الـ ٣ الماضية:		
L2	هل حاولت التحكم في وزنك أو منع زيادة وزنك أو تقييد كمية الطعام المستهلكة، على الرغم من وزنك المنخفض؟	نعم	لا ←
L3	هل كنت تخشى بشدة من زيادة الوزن أو السمنة، على الرغم من أن وزنك أقل من المعدل الطبيعي؟	نعم	لا ←
L4. a	هل اعتبرت نفسك كبير الحجم / سميناً أو أن جزء من جسمك كبير الحجم / سمين؟	نعم	لا
L4. b	هل أثر وزنك أو شكل جسمك بشكل كبير على شعورك تجاه نفسك؟	نعم	لا
L4. c	هل كنت تعتقد أن وزن جسمك الحالي (المنخفض) عادي أو زائد؟	نعم	لا
L5	**هل تم تسجيل إجابة "نعم" في واحد أو أكثر من أسئلة البند L4؟**	نعم	لا ←
	**هل تم تسجيل إجابة "نعم" للبند L5؟**	نعم	لا
	فقدان شهية عصبي حالي<br>ANOREXIA NERVOSA<br>CURRENT		

© 2019 Charité Universitätsmedizin Berlin - Campus Benjamin Franklin. All rights reserved. Translated by Dana Churbaji, Shaymaa Hauary, Sara Mohamed & Carine Karnouk. Supervised by Dr. Malek Bajbouj.

جدول الطول/الوزن متسق مع الحد الأدنى لمقياس كتلة الجسم للشخص البالغ ١٧كغ لكل متر مربع

جدول الطول مقابل الوزن الموافق لمقياس الـ BMI لـ١٧كغ لكل متر مربع

الطول / الوزن

cm	145	147	150	152	155	158	160	163	165	168	170	173	175	178
kg	37	38	39	41	42	43	45	46	48	49	51	52	54	55

cm	180	183	185	188	191
kg	57	59	60	62	64

يتم حساب عتبات الوزن أعلاه باستخدام مؤشر كتلة الجسم (BMI) ما يساوي أو يقل عن 17.0 كجم / للمتر المربع لارتفاع المريض باستخدام مقياس الـ BMI لمركز التحكم في الأمراض والوقاية منها. العتبات المدونة هنا تعتبر مقياس لتشخيص المريض بنقصان الوزن مبنياً على الـ DSM-5 لاضطراب فقدان الشهية العصبي.

© 2019 Charité Universitätsmedizin Berlin - Campus Benjamin Franklin. All rights reserved. Translated by Dana Churbaji, Shaymaa Hauary, Sara Mohamed & Carine Karnouk. Supervised by Dr. Malek Bajbouj.

الشره العصبي .M
BULIMIA NERVOSA

هذا السهم ← يعني انتقل إلى مربع التشخيص النهائي الخاص بالوحدة واختر (لا) في مربع تشخيص الشره العصبي وفي مربع تشخيص فقدان الشهية العصبي (نمط الأكل بشراهة Binge eating /نمط التطهير Purging (تناول مسهلات أو الترجيع). لكن إذا تم تسجيل استجابة إيجابية في مربع التشخيص على مرض فقدان الشهية العصبي (في الوحدة L) ، فاستمر في الإجابة على الأسئلة بصرف النظر عن السهم لتتمكن من تسجيل أو تحديد نوع أو نمط فقدان الشهية العصبي)

M1	**خلال الأشهر الثلاثة الماضية**، هل انتابتك نوبات من الشراهة أو كانت هناك أوقات تناولت فيها كمية كبيرة جدًا من الطعام خلال ساعتين؟	نعم	لا ←
M2	خلال هذه النوبات، هل شعرت أن تناولك للطعام كان خارج عن السيطرة؟	نعم	لا ←

	**خلال الأشهر الثلاثة الماضية:**		
M3	هل كانت تنتابك نوبات شراهة بمعدل يصل إلى مرة واحدة في الأسبوع؟	نعم	لا ←
M4	هل فعلت أي شيء لكي تعوض أو تمنع زيادة الوزن الناتج عن نوبات الشراهة، مثل التقيؤ، الصيام، ممارسة الرياضة أو تناول أدوية مُسهلة ، أو استخدام الحقن الشرجية ، أو تناول مدرات البول (حبوب منع احتباس السوائل) ، أو تناولت أدوية أخرى؟ هل تفعل هذا بمعدل مرة واحدة في الأسبوع؟	نعم	لا ←
M4 a.	عدد نوبات السلوك التعويضي غير المناسبة، في الأسبوع؟		
	عدد الأيام التي تتم فيها السلوكيات التعويضية غير المناسبة في الأسبوع؟		
M5	هل يؤثر وزنك أو شكل جسمك بشكل كبير على شعورك تجاه نفسك؟	نعم	لا ←
M6	**هل تفي أعراض المريض بمعايير تشخيص فقدان الشهية العصبي؟**	نعم	لا ← to M8
M7	هل حدثت نوبات الشراهة عندما كان وزنك أقل من (______ رطل / كجم) فقط؟		
	**القائم على إجراء المقابلة: اكتب بين القوسين أعلاه الحد الأدنى لوزن المريض نسبة لطوله من جدول الطول/الوزن في وحدة فقدان الشهية العصبية**		

M8	**هل تم تسجيل إجابة "نعم" في البند M5؟**	نعم	لا ←
	**إضافة إلى تسجيل إجابة "لا" في أحد بنود M6 أو M7؟**		
	الشره العصبي (حالي)<br>BULIMIA NERVOSA<br>CURRENT		

	**هل تم تسجيل إجابة "نعم" في البند M7؟**	نعم	لا ←
	فقدان الشهية العصبي<br>(نمط الأكل بشراهة /نمط التطهير (تناول مسهلات أو التقيؤ)<br>حالي<br>BULIMIA NERVOSA<br>Binge Eating/Purging Type<br>CURRENT		

© 2019 Charité Universitätsmedizin Berlin - Campus Benjamin Franklin. All rights reserved. Translated by Dana Churbaji, Shaymaa Hauary, Sara Mohamed & Carine Karnouk. Supervised by Dr. Malek Bajbouj.

	**هل تفي أعراض المريض بمعايير تشخيص فقدان الشهية العصبي؟**<br>**و هل تم تسجيل إجابة "لا" في البند M2 و M4؟**	نعم	لا<br>←
فقدان الشهية العصبي<br>نمط تقييدي (استخدام حمية غذائية) حالي<br>ANOREXIA NERVOSA<br>Restricting Type<br>CURRENT			

محددات شدّة اضطراب الأكل
خفيف = من 1 - 3 نوبات من السلوكيات التعويضية غير المُناسبة
متوسط = من 4 -7 نوبات من السلوكيات التعويضية غير المُناسبة
شديد = من 8 - 11 نوبة من السلوكيات التعويضية غير المُناسبة
بالغة الشدة = 14 نوبة أو أكثر من السلوكيات التعويضية غير المُناسبة

حدد شدة الاضطراب			
خفيف<br>☐	متوسط<br>☐	شديد<br>☐	بالغة الشدة<br>☐

© 2019 Charité Universitätsmedizin Berlin - Campus Benjamin Franklin. All rights reserved. Translated by Dana Churbaji, Shaymaa Hauary, Sara Mohamed & Carine Karnouk. Supervised by Dr. Malek Bajbouj.

اضطراب نوبات الأكل الشره .MB
BINGE EATING DISORDER

هذا السهم ← يعني انتقل إلى مربع التشخيص النهائي الخاص بالوحدة واختر (لا) في مربع التشخيص. انتقل بعدها إلى الوحدة التالية.

لا	نعم ←	**هل تفي أعراض المريض بمعايير تشخيص فقدان الشهية العصبي؟**	MB1
لا	نعم ←	**هل تفي أعراض المريض بمعايير تشخيص الشره العصبي؟**	MB2
لا ←	نعم	**هل تم تسجيل إجابة "نعم" في البند M2؟**	MB3

لا ←	نعم	**هل تم تسجيل إجابة "نعم" في البند M3؟**	MB4
لا	نعم ←	**هل تم تسجيل إجابة "نعم" في البند M4؟**<br>**إذا تم تجاوز البند M4 في الوحدة M-الشره العصبي قم بتوجيه السؤال الخاص بالبند M4 الآن لكي تتمكن من الإجابة على البند MB5**	MB5

		<u>خلال الأشهر الثلاثة الماضية:</u>	
لا	نعم	هل أكلت بسرعة أكبر عن من المعتاد؟	MB6. a
لا	نعم	هل أكلت حتى شعرت بامتلاء شديد وغير مريح؟	MB6. b
لا	نعم	هل تناولت كميات كبيرة من الطعام عند عدم شعورك بالجوع؟	MB6. c
لا	نعم	هل كنت تتناول الطعام بمفردك لأنك كنت تشعر بالحرج من كمية الطعام التي كنت تتناولها؟	MB6. d
لا	نعم	هل شعرت بالذنب أو بالاكتئاب أو بالاشمئزاز من نفسك بعد نوبة الشره؟	MB6. e
لا ←	نعم	**هل تم تسجيل إجابة "نعم" في ٣ أو أكثر من أسئلة البند MB6؟**	
لا ←	نعم	هل تسبب لك نوبات الشره المعاناة بشكل كبير؟	MB7
		عدد نوبات الشره في الأسبوع؟ ______	MB8
		عدد الأيام التي يحدث فيها الشره في الأسبوع؟_______	
لا	***نعم***	**هل تم تسجيل إجابة "نعم" في البند MB7؟**	
		اضطراب نوبات الأكل الشره<br>BINGE-EATING DISORDER<br>CURRENT	

محددات شدّة اضطراب الأكل
خفيف = من 1 - 3 نوبات من السلوكيات التعويضية غير المُناسبة
متوسط = من 4 -7 نوبات من السلوكيات التعويضية غير المُناسبة

© 2019 Charité Universitätsmedizin Berlin - Campus Benjamin Franklin. All rights reserved. Translated by Dana Churbaji, Shaymaa Hauary, Sara Mohamed & Carine Karnouk. Supervised by Dr. Malek Bajbouj.

شديد = من 8 – 13 نوبة من السلوكيات التعويضية غير المُناسبة
بالغة الشدة = 14 نوبة أو أكثر من السلوكيات التعويضية غير المُناسبة

حدد شدة الاضطراب			
خفيف<br>☐	متوسط<br>☐	شديد<br>☐	بالغة الشدة<br>☐

© 2019 Charité Universitätsmedizin Berlin - Campus Benjamin Franklin. All rights reserved. Translated by Dana Churbaji, Shaymaa Hauary, Sara Mohamed & Carine Karnouk. Supervised by Dr. Malek Bajbouj.

اضطراب القلق العام .N
GENERALIZED ANXIETY DISORDER

هذا السهم ← يعني انتقل إلى مربع التشخيص النهائي الخاص بالوحدة واختر (لا) في مربع التشخيص.
انتقل بعدها إلى الوحدة التالية.

N1. a	هل كنت قلقاً بشكل مفرط أو مهموماً بشأن العديد من الأشياء الروتينية خلال الأشهر الـ6 الماضية؟	نعم	لا ←
	**في حال عدم فهم المريض ما تعنيه, يمكنك الإيضاح عن طريق السؤال التالي مع ذكر أمثلة** هل يعتقد الآخرون بأنك شخص قَلِق أو نموذج للـ" الشخص الذي يتوتر ويقلق على أشياء غير مُبررة وينشر جو من القلق والتوتر بين الناس "Worrywart"؟		
N1. b	هل هذا القلق موجود في معظم الأيام؟	نعم	لا ←
	**هل القلق الخاص بالمريض مُقتصر بشكل حصري على، أو يُمكن تفسيره بشكل أفضل بواسطة، أي اضطراب سابق لهذا الجزء من المقابلة؟**	نعم ←	لا

N2	هل تجد صعوبة في السيطرة على هذا القلق؟	نعم	لا ←
N3	**قم بتسجيل إجابة "لا" على الأسئلة القادمة لو كانت الأعراض مقتصرة على أي اضطراب سابق أو قد قُمت بالاستفسار عنه قبل هذا الجزء من المقابلة.** عندما كنت قلقًا خلال الـ ٦ أشهر الماضية، هل كنت في أغلب الوقت:		
a.	تشعر بالقلق, أو الانقباض أو الانفعال؟	نعم	لا
b.	هل كان لديك تشنج في العضلات؟	نعم	لا
c.	تشعر بالتعب، أو الضعف، أو تُصاب بالإرهاق بسهولة؟	نعم	لا
d.	تواجه صعوبة في التركيز أو تجد عقلك فارغًا؟	نعم	لا
e.	تشعر أنك منفعل (سريع الغضب)؟	نعم	لا
f.	تواجه صعوبات في النوم (صعوبة في الخلود إلى النوم, أو الاستيقاظ في منتصف الليل أو الاستيقاظ في الصباح الباكر أو النوم بشكل مفرط)؟	نعم	لا
	**هل تم تسجيل إجابة "نعم" في ٣ أسئلة أو أكثر من البند N3؟**	نعم	لا ←
N4	هل تسبب هذا القلق في تعطيل قدرتك على العمل أو التفاعل على المستوى الاجتماعي أو أثر على علاقاتك بالآخرين أو أثر على مجالات أخرى مهمة في حياتك أو تسبب لك في معاناة ملحوظة؟	***نعم***	لا
	**هل تم تسجيل إجابة "نعم" على البند الخاص بـ "استبعاد السبب العضوي" التابع للملخص O2؟**		
	اضطراب القلق العام – حالي<br>GENERALIZED ANXIETY DISORDER CURRENT		

© 2019 Charité Universitätsmedizin Berlin - Campus Benjamin Franklin. All rights reserved. Translated by Dana Churbaji, Shaymaa Hauary, Sara Mohamed & Carine Karnouk. Supervised by Dr. Malek Bajbouj.

استبعاد الأسباب الطبيبة، العضوية، المخدرات/العقاقير لجميع الاضطرابات .O
RULE OUT MEDICAL, ORGANIC OR DRUG CAUSES FOR ALL DISORDERS

في حال تسجيل "نعم" في مربع تشخيص أي اضطراب حالي أو نوبة اكتئاب كبرى أو نوبة هوس أو نوبة هوس خفيف, اسأل التالي:

	**قبل ظهور هذه الأعراض مباشرة:**			
O1.a	هل كنت تتناول أي أدوية/عقاقير أو مواد مخدرة أو كنت تمر بأعراض انسحاب من أي منها؟	غير مؤكد	نعم	لا
O1.b	هل تُعاني من أي مرض؟	غير مؤكد	نعم	لا
O2	**إذا تم تسجيل "نعم" في أي من الأسئلة O1.a أو O1.b;**<br><br>**برأي القائم بإجراء المقابلة، هل من المحتمل أن يكون أي من هذه الأمراض سبباً مباشراً في تشخيص اضطرابات المريض؟ إذا لزم الأمر، اسأل أسئلة إضافية مفتوحة.**	غير مؤكد	نعم	لا
O2 summary	**هل تم استبعاد الأسباب العضوية/الطبية/ المرتبطة بالمخدرات/العقاقير؟**<br>**إذا تم تسجيل إجابة "نعم" في ;O2 فإن إجابة ملخص O2 تكون "لا."**<br>**إذا تم تسجيل إجابة "لا" في ;O2 فإن إجابة ملخص O2 تكون "نعم."**<br>**خلاف ذلك، حدد "غير مؤكد" في ملخص O2**	غير مؤكد	نعم	لا

© 2019 Charité Universitätsmedizin Berlin - Campus Benjamin Franklin. All rights reserved. Translated by Dana Churbaji, Shaymaa Hauary, Sara Mohamed & Carine Karnouk. Supervised by Dr. Malek Bajbouj.

P. اضطراب الشخصية المعادية للمجتمع
ANTISOCIAL PERSONALITY DISORDER

هذا السهم ← يعني انتقل إلى مربع التشخيص النهائي الخاص بالوحدة واختر (لا) في مربع التشخيص. انتقل بعدها إلى الوحدة التالية.

P1	قبل بلوغك سن الـ١٥, هل:		
a.	كنت تهرب من المدرسة أو تهرب من البيت طوال الليل بصفة متكررة, أو كنت تتأخر ليلاً في العودة إلى المنزل بما يتنافى مع قواعد والديك في المنزل؟	نعم	لا
b.	كنت تكذب، تغش، تخدع الآخرين، أو تسرق بصفة متكررة, أو سرقت أو اقتحمت منزل شخص ما أو سيارته؟	نعم	لا
c.	هل كنت تبادر بافتعال المشاكل أو الخناق أو البلطجة، أو بتهديد ، أو بتخويف الآخرين؟	نعم	لا
d.	هل قمُت بتدمير الأشياء عمداً أو قُمت بإشعال الحرائق؟	نعم	لا
e.	تعمدت إلحاق الأذى بالأشخاص أو بالحيوانات؟	نعم	لا
f.	أجبرت شخصاً على ممارسة الجنس معك؟	نعم	لا
	**هل تم تسجيل إجابة "نعم" على ٢ أو أكثر من أسئلة البند P1؟**	نعم	لا ←
	**لا تقم بتسجيل إجابة السلوكيات الموضحة أدناه بـ نعم إذا كانت دوافعها مقتصرة على خلفيات سياسية أو دينية.**		
P2	منذ بلوغك سن الـ١٥ سنة:		
a.	هل سبق و قُمت بأفعال غير قانونية أو بأشياء يُمكن أن تكون سبباً في القبض عليك، حتى لو لم يتم القبض عليك (على سبيل المثال، تدمير الممتلكات، أو السرقة، أو سرقة سلع من المتجر، أو بيع الأدوية/المُخدرات، أو ارتكاب مخالفات قانونية جديّة)؟	نعم	لا
b.	هل سبق وكذبت أو "خدعت" أشخاص آخرين للحصول على المال أو المتعة، أو كذبت لمجرد التسلية؟	نعم	لا
c.	هل سبق وأن كنت متسرعاً ومندفعًا ولم تهتم بالتخطيط المسبق؟	نعم	لا
d.	هل سبق وأن دخلت في عراك جسدي بشكل متكرر أو قُمت بالهجوم والتعدي على الآخرين (بما في ذلك عراك جسدي مع زوجتك أو أطفالك)؟	نعم	لا
e.	هل سبق وعرضت نفسك أو الآخرين للخطر دون مبالاة؟	نعم	لا
f.	هل سبق أن تصرفت بشكل متكرر بطريقة يعتبرها الآخرون غير مسؤولة, كعدم القدرة على دفع مبالغ مترتبة عليك, أو التصرّف بشكل اندفاعي عمداً, أو أخذ قرار بعدم العمل لإعالة نفسك؟	نعم	لا
g.	لا تشعر بالذنب بعد القيام بإيذاء الآخرين، أو إساءة معاملة الآخرين أو الكذب أو السرقة أو تحطيم ممتلكاتهم؟	نعم	لا
	**هل تم تسجيل إجابة "نعم" على ٢ أو أكثر من أسئلة البند P2؟**	***نعم***	لا
	اضطراب الشخصية المعادية للمجتمع – مزمن مدى الحياة<br>ANTISOCIAL PERSONALITY DISORDER LIFETIME		

© 2019 Charité Universitätsmedizin Berlin - Campus Benjamin Franklin. All rights reserved. Translated by Dana Churbaji, Shaymaa Hauary, Sara Mohamed & Carine Karnouk. Supervised by Dr. Malek Bajbouj.

اضطرابات المزاج: نظام التشخيص

الوحدات:	A	نوبة الاكتئاب الكبرى
	C	(نوبة الهوس الخفيف) نوبة الهوس
	K	الاضطرابات الذهانية

الوحدة K			
1a	**هل تم تسجيل إجابة نعم في K11b ؟**	**نعم**	**لا**
1b	**هل تم تسجيل إجابة نعم في K12a ؟**	**نعم**	**لا**

الوحدتان A و C:		حالي	ماضي
2a	ضع دائرة حول "**نعم**" إذا تم تحديد فكرة ضلالية في **A3e**<br>أو في حالة وجود أي ملامح ذهانية في **K1** إلي **K7**	**نعم**	**لا**
2b	ضع دائرة حول "**نعم**" إذا تم تحديد فكرة ضلالية في **C3a**<br>أو في حالة وجود أي ملامح ذهانية في **K1** إلي **K7**	**نعم**	**لا**
2c	**هل تم تسجيل نوبة الاكتئاب الكبرى بـ "نعم" (حالية أو ماضية)؟**<br>**و**<br>**هل تم تسجيل نوبة الهوس بـ "لا" (حالية أو ماضية)؟**<br>**و**<br>**هل تم تسجيل نوبة الهوس الخفيف بـ "لا" (حالية أو ماضية)؟**<br>**و**<br>**هل تم تسجيل إجابة "نعم" على البند الخاص بـ "استبعاد السبب العضوي" التابع للملخص O2؟**<br>**حدد:**<br>• **إذا كانت نوبة الاكتئاب حالية أو ماضية أو كليهما**<br>• **إذا كانت مع ملامح ذهانية، حالية: إذا كانت إجابة 1b أو 2a (حالية) = نعم**<br>**أو مع ملامح ذهانية، ماضية: إذا كانت إجابةa1 أو a2 (ماضية) = نعم**		

اضطراب الاكتئاب الرئيسي	
□ ماضي	□ حالي
اضطراب الاكتئاب الرئيسي مع ملامح ذهانية	
□ ماضي	□ حالي

© 2019 Charité Universitätsmedizin Berlin - Campus Benjamin Franklin. All rights reserved. Translated by Dana Churbaji, Shaymaa Hauary, Sara Mohamed & Carine Karnouk. Supervised by Dr. Malek Bajbouj.

d	هل تم تسجيل نوبة الهوس بـ **"نعم"** (حالية أو ماضية)؟<br>**و**<br>هل تم تسجيل إجابة **"نعم"** على البند الخاص بـ "استبعاد السبب العضوي" التابع للملخص **O2**؟<br>**حدد:**<br>• إذا كان اضطراب ثنائي القطب النوع الأول **حالي** أو **ماضي** أو كليهما<br>• أو مع نوبة هوس أحُادية:<br>إذا كانت نوبة الهوس (حالية أو ماضية) = **نعم**<br>**وإذا كانت نوبة** الاكتئاب الكبرى (حالية وماضية) = **لا**<br><br>• أو إذا كان مع وجود ملامح ذهانيه، حالية: إذا كانت الإجابة على **b1** أو **a2** (حالية)أو **2b** (حالية) = **نعم**<br>مع وجود ملامح ذهانية، ماضية: إذا كانت الإجابة على **a1** أو **a2** (ماضية) أو **2b** (ماضية) = **نعم**<br>• إذا كانت النوبة الأخيرة هي نوبة هوس، أو نوبة اكتئاب، أو نوبة هوس خفيف (يستبعد كل منهما الآخر)<br>• إذا كانت نوبة المزاج الأخيرة مختلطة، أي مع وجود قلق وملامح ذهانية<br>هوس خفيف/هوس مع ملامح مُختلطة = هوس خفيف/هوس + ما لا يقل عن **3** أعراض من **A3**<br>اكتئاب مع ملامح مُختلطة = نوبة اكتئاب كبرى + ما لا يقل عن **3** أعراض من **C3**<br>مع وجود قلق = مع ما لا يقل عن **3** أعراض من **N3**

**اضطراب ثنائي القطب النوع الأول**		
	**حالي**	**ماضي**
**اضطراب ثنائي القطب نوع أول**	☐	☐
**نوبة هوس أحادية**	☐	☐

مع ملامح ذهانية
☐ حالية
☐ ماضية

أخر نوبة
☐ هوس
☐ اكتئاب
☐ هوس خفيف

أخر نوبة
☐ مع ملامح مختلطة
☐ مع قلق

أخر نوبة
☐ خفيفة
☐ معتدل
☐ شديدة

© 2019 Charité Universitätsmedizin Berlin - Campus Benjamin Franklin. All rights reserved. Translated by Dana Churbaji, Shaymaa Hauary, Sara Mohamed & Carine Karnouk. Supervised by Dr. Malek Bajbouj.

e

هل تم تسجيل نوبة الاكتئاب الكبرى بـ "**نعم**" (حالية أو ماضية)؟

و

هل تم تسجيل نوبة الهوس الخفيف بـ "**نعم**" (حالية أو ماضية)؟

و

هل تم تسجيل نوبة الهوس بـ "**لا**" (حالية أو ماضية)؟

و

هل تم تسجيل إجابة "**نعم**" على البند الخاص بـ "استبعاد السبب العضوي" التابع للملخص **O2**؟

حدد:

- إذا كان اضطراب ثنائي القطب **النوع الثاني حالي** أو **ماضي** أو كليهما
- إذا كانت أخر نوبة مزاج هي نوبة هوس خفيف أو اكتئاب (يستبعد كل منهما الآخر)
- إذا كانت آخر نوبة مزاج مع ملامح مختلطة أو مع قلق فقط أو مع ملامح ذهانية فقط

هوس خفيف مع وجود ملامح مختلطة = هوس خفيف + ما لا يقل عن **3** أعراض من **A3**

اكتئاب مع وجود ملامح مختلطة = نوبة اكتئاب كبري + ما لا يقل عن **3** أعراض من **C3**

مع وجود قلق = مع ما لا يقل عن **3** أعراض من **N3**

اضطراب ثنائي القطب النوع الثاني

	ماضي	حالي
**اضطراب ثنائي القطب النوع الثاني**	☐	☐

أخر نوبة

☐ هوس خفيف

☐ اكتئاب

أخر نوبة

☐ مع ملامح مختلطة

☐ مع قلق

أخر نوبة

☐ خفيفة

☐ معتدل

☐ شديدة

© 2019 Charité Universitätsmedizin Berlin - Campus Benjamin Franklin. All rights reserved. Translated by Dana Churbaji, Shaymaa Hauary, Sara Mohamed & Carine Karnouk. Supervised by Dr. Malek Bajbouj.

f	هل تم تسجيل نوبة الاكتئاب الكبرى بـ **"لا"** (حالية وماضية)؟<br>**و**<br>هل تم تسجيل نوبة الهوس بـ **"لا"** (حالية وماضية)؟<br>**و**<br>هل تم الإجابة على **C4b** بـ **"نعم"** للإطار الزمني المناسب؟<br>**و**<br>هل تمت الإجابة على **C8b بـ "نعم"**؟<br>**او**<br>هل تم تسجيل نوبة الهوس بـ **"لا"** (حالية وماضية)؟<br>**و**<br>هل تم تسجيل نوبة الهوس الخفيف بـ **"لا"** (حالية وماضية)؟<br>**و**<br>هل تمت الإجابة على **C4a** بـ **"نعم"** للإطار الزمني المناسب؟<br>**و**<br>هل تمت الإجابة على **C8c بـ "نعم"**؟<br><br>حدد ما إذا كانت الاضطرابات ثنائية القطب الأخرى المحددة وذات الصلة<br>(Other Specified Bipolar and Related Disorder)<br>حالية أو **ماضية** أو كليهما.

**اضطرابات ثنائي القطب الأخرى المحددة وذات الصلة**		
	**ماضي**	**حالي**
**اضطرابات ثنائي القطب الأخرى المحددة وذات الصلة**	☐	☐

© 2019 Charité Universitätsmedizin Berlin - Campus Benjamin Franklin. All rights reserved. Translated by Dana Churbaji, Shaymaa Hauary, Sara Mohamed & Carine Karnouk. Supervised by Dr. Malek Bajbouj.

قياسات التقييم الاختياري لتتبُع التغييُرات مع مرور الوقت
OPTIONAL ASSESSMENT MEASURES TO TRACK CHANGES OVER TIME

A. أسئلة عامّة

شدة الأعراض

استخدم هذا المقياس لتقييم شدة الأعراض الخاص بك وقُم بتسجيل النتيجة في العمود المخصص لذلك في الجدول أدناه:

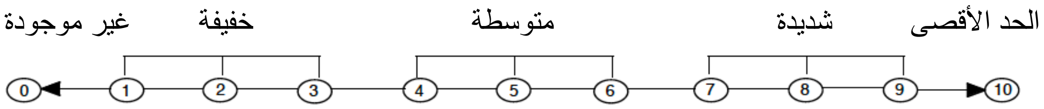

	تقييم الأعراض المشتركة بين الاضطرابات	التقييم
1	اكتئاب	
2	غضب	
3	هوس (الشعور بحالة من " النشوة " أو " الابتهاج " أو "النشاط الزائد والاهتياج" أو الامتلاء بالطاقة أو الحيوية المفرطة مع وجود أفكار مُتسارعة)	
4	قلق	
5	الأعراض البدنية (الجسمية)	
6	أفكار انتحارية أو دوافع مُفاجئة/مُلحة/قوية أو خطط أو نوايا (أي أفكار لقتل نفسك) أو أي استعدادات لقتل نفسك أو أي محاولة لقتل نفسك	
7	سماع أصوات (من أشياء أو من أشخاص) لا يمكن للآخرين سماعها أو الخوف من قدرة شخص ما على سماع أو قراءة أفكارك أو الاعتقاد بأشياء لا يتقبلها الآخرون كحقيقة (على سبيل المثال أن الناس يتجسسون عليك أو يتآمرون عليك أو يتحدثون عنك (الذهان))	
8	مشاكل/اضطرابات النوم	
9	مشاكل الذاكرة	
10	أفكار وسواسية أو تكرارية أو سلوكيات قهرية	
11	اختلاف إدراكك للأمور المحيطة بك كأنها غير مألوفة, غريبة, أو بأنك خرجت من جسدك أو انعزلت عنه كاملا أو عن جزء منه؟	
12	القدرة على الأداء في العمل، في المنزل ، في حياتك ، أو في علاقاتك	
13	الإفراط في تناول الكحول أو المخدرات/العقاقير	

© 2019 Charité Universitätsmedizin Berlin - Campus Benjamin Franklin. All rights reserved. Translated by Dana Churbaji, Shaymaa Hauary, Sara Mohamed & Carine Karnouk. Supervised by Dr. Malek Bajbouj.

B. العجز/ضعف الأداء

شدة الضعف/العجز

استخدم هذا المقياس لتقييم الي أي مدي عطلت أعراضك المجالات التالية من حياتك: قم بتسجيل النتيجة في العمود المخصص لذلك في الجدول أدناه:

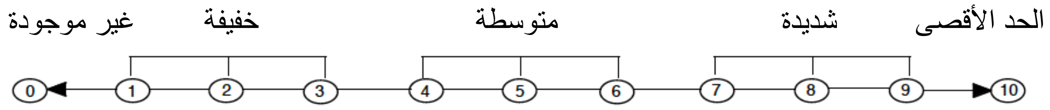

	تقييم شدة الضعف/العجز	التقييم
1	العمل أو الواجبات المدرسية	
2	الحياة الاجتماعية أو الأنشطة الترفيهية (مثل الهوايات أو الأشياء التي تقوم بها للاستمتاع)	
3	الحياة الأسرية و / أو المسؤوليات المنزلية	
4	القدرة على التوافق/التكيف/التعامل مع الناس	
5	العلاقات الشخصية والاجتماعية	
6	القدرة على الفهم والتواصل مع الآخرين	
7	القدرة على الاعتناء بنفسك (الاغتسال/التشطيف، الاستحمام، دخول الحمام لقضاء الحاجه، ارتداء الملابس بشكل صحيح، تنظيف الأسنان بالفرشاة ، الغسيل ، تمشيط / تنظيف الشعر ، تناول الطعام بانتظام)	
8	التخريب أو العدوانية تجاه الآخرين	
9	مالياً (القدرة على إدارة أموالك)	
10	القدرة "البدنية" على الحركة والتجول	
11	الحياة الروحانية أو الدينية	
12	ما مدى تأثير حالتك على الآخرين في أسرتك؟	

© 2019 Charité Universitätsmedizin Berlin - Campus Benjamin Franklin. All rights reserved. Translated by Dana Churbaji, Shaymaa Hauary, Sara Mohamed & Carine Karnouk. Supervised by Dr. Malek Bajbouj.

REFERENCES

1. Sheehan DV, Lecrubier Y, Harnett-Sheehan K, Amorim P, Janavs J, Weiller E, Hergueta T, Baker R, Dunbar G: The Mini International Neuropsychiatric Interview (M.I.N.I.): The Development and Validation of a Structured Diagnostic Psychiatric Interview. J. Clin Psychiatry, 1998;59(suppl 20): 22-33.

2. Sheehan DV, Lecrubier Y, Harnett-Sheehan K, Janavs J, Weiller E, Bonara LI, Keskiner A, Schinka J, Knapp E, Sheehan MF, Dunbar GC. Reliability and Validity of the MINI International Neuropsychiatric Interview (M.I.N.I.): According to the SCID-P. European Psychiatry. 1997; 12:232-241.

3. Lecrubier Y, Sheehan D, Weiller E, Amorim P, Bonora I, Sheehan K, Janavs J, Dunbar G. The MINI International Neuropsychiatric Interview (M.I.N.I.) A Short Diagnostic Structured Interview: Reliability and Validity According to the CIDI. European Psychiatry. 1997; 12: 224-231.

4. Amorim P, Lecrubier Y, Weiller E, Hergueta T, Sheehan D: DSM-III-R Psychotic Disorders: procedural validity of the Mini International Neuropsychiatric Interview (M.I.N.I.). Concordance and causes for discordance with the CIDI. European Psychiatry. 1998; 13:26-34.

ACKNOWLEDGEMENTS

The author wishes to acknowledge the valuable contributions made to the earlier versions of the MINI for DSM III-R and DSM IV by:

1. Yves Lecrubier, my close collaborator (now deceased) on the initial development of the MINI for DSM III-R, the DSM IV and ICD-10.
2. Juris Janavs, Emanuelle Weiller, Christer Allgulander, Kathy Harnett-Sheehan, Roxy Baker, Michael Sheehan, Chris Gray, Thierry Hergueta, N. Kadri, David Baldwin, Christian Even, Rosario Hidalgo, Marelli Soto-Colon, Ossama Osman.
3. Patricia Amorim for her extensive work on the development of the expanded version of the Psychotic Disorders Module and algorithms for DSM III-R. We have evolved her model further in the MINI for Psychotic Disorders 7 and in the MINI Plus 7 for DSM-5.
4. Executive Scientific committee for the MINI 6.0.0 for DSM-IV:
   Christer Allgulander, Stockholm, Sweden
   A. Carlo Altamura, Milano, Italy
   Cyril Hoschl, Praha, Czech Republic
   George Papadimitriou, Athens, Greece
   Hans Ågren, Göteborg, Sweden
   Hans-Jürgen Möller, München, Germany
   Hans-Ulrich Wittchen, Dresden, Germany
   István Bitter, Budapest, Hungary
   Jean-Pierre Lépine, Paris, France
   Jules Angst, Zurich, Switzerland
   Julio Bobes, Oviedo, Spain
   Luciano Conti, Pisa, Italy
   Marelli Colon-Soto MD, Puerto Rico, United States
   Michael Van Ameringen MD, Toronto, Canada

© 2019 Charité Universitätsmedizin Berlin - Campus Benjamin Franklin. All rights reserved. Translated by Dana Churbaji, Shaymaa Hauary, Sara Mohamed & Carine Karnouk. Supervised by Dr. Malek Bajbouj.

Rosario Hidalgo MD, Tampa, United States
Siegfried Kasper, Vienna, Austria
Thomas Schlaepfer, Bonn, Germany

5. Mapi and the many academic translation teams internationally who collaborated in ensuring that quality translations became available in over 70 languages or language variants. Mapi (http://www.mapigroup.com) is now the official translation and linguistic validation service for all variants of the MINI.
6. Individual clinicians and patients who over the years made countless suggestions to help improve the accuracy and clinical value to the MINI: JM Giddens for her advice on the MINI 7 version of the Suicidality Disorders Module, Dr. Michael Van Ameringen for assistance with the ADHD module, and Dr Pauline Powers for her advice on the modules on Anorexia Nervosa and Bulimia.
7. Validation studies of the MINI were made possible, in part, by grants from SmithKlineBeecham and the European Commission.

© 2019 Charité Universitätsmedizin Berlin - Campus Benjamin Franklin. All rights reserved. Translated by Dana Churbaji, Shaymaa Hauary, Sara Mohamed & Carine Karnouk. Supervised by Dr. Malek Bajbouj.
